# Striosome-based map of the mouse striatum that is conformable to both cortical afferent topography and uneven distributions of dopamine D1 and D2 receptor-expressing cells

**DOI:** 10.1007/s00429-018-1749-3

**Published:** 2018-09-10

**Authors:** Yuta Miyamoto, Sachiko Katayama, Naoki Shigematsu, Akinori Nishi, Takaichi Fukuda

**Affiliations:** 1 Department of Anatomy and Neurobiology, Graduate School of Medical Sciences, Kumamoto University, 1-1-1 Honjo, Chuo-ku, Kumamoto, 860-8556 Japan; 2 Department of Pharmacology, Kurume University, Kurume, 830-0111 Japan

**Keywords:** Striatum, Striosome, Dopamine receptor, Substance P, µ-Opioid receptor, Enkephalin

## Abstract

The striatum is critically involved in execution of appropriate behaviors, but its internal structures remain unmapped due to its unique structural organization, leading to ambiguity when interpreting heterogeneous properties of striatal neurons that differ by location. We focused on site-specific diversity of striosomes/matrix compartmentalization to draw the striatum map. Five types of striosomes were discriminated according to diverse immunoreactivities for the µ-opioid receptor, substance P (SP) and enkephalin, and each type occupied a particular domain inside the striatum. Furthermore, there was an additional domain lacking striosomes. This striosome-free space was located at the dorsolateral region and received afferents preferentially from the primary motor and sensory cortices, whereas the striosome-rich part received afferents from associational/limbic cortices, with topography inside both innervations. The proportion of dopamine D1 receptor-expressing, presumptive striatonigral neurons was approximately 70% in SP-positive striosomes, 40% in SP-deficient striosomes, 30% in the striosome-free space, and 50% in the matrix. In contrast, the proportion of D2 receptor-expressing, presumptive striatopallidal neurons was complementary to that of D1 receptor-expressing cells, indicating a close relationship between the map and the direct and indirect parallel circuitry. Finally, the most caudal part of the striatum lacked compartmentalization and consisted of three lamina characterized by intense and mutually exclusive immunoreactivities for SP and enkephalin. This tri-laminar part also received specific afferents from the cortex. The newly obtained map will facilitate broad fields of research in the basal ganglia with higher resolution of the three-dimensional anatomy of the striatum.

**Electronic supplementary material:**

The online version of this article (10.1007/s00429-018-1749-3) contains supplementary material, which is available to authorized users.

## Introduction

The striatum is the major input structure of the basal ganglia and plays pivotal roles in optimizing behaviors through learning. It occupies a large portion of the subcortical gray matter in the mammalian telencephalon and receives driving inputs from the entire neocortex. These anatomical characteristics indicate versatile functions of the striatum such as control of voluntary movements, goal-directed behaviors, decision-making, cognition, and habituation (Graybiel 2008; Pennartz et al. 2009; Crittenden and Graybiel 2011). However, in contrast with other brain areas, the internal structure of the striatum has been difficult to depict as a map because its complex architecture appears to be organized under unknown principles that are quite different from those of other areas. Thus, neither laminar nor columnar organization, which are generally seen in cortical tissues, can be applied to the striatum. Further attempts to classify the striatum into several subnuclei, as done in the thalamus, have also not been successful.

Alternatively, functional division of the striatum into sensorimotor, associational, and limbic parts has been adopted in primates (Parent 1990). Analogous classifications have also been assigned to the rodent striatum (McGeorge and Faull 1989; Voorn et al. 2004; Graybiel 2008). However, in both cases, the anatomical positions of these divisions are only roughly described, e.g., the dorsolateral part in rodent striatum is sensorimotor, the dorsomedial part is associational, and the ventral part is the limbic division. Because information regarding the actual positions of these divisions is not currently available, they are only conceptually presented in schematic images of the striatum. Thus, even researchers using the newest technologies still do not have a common reference map that can be shared with others to facilitate mutual understanding of experimental data such as site-specific expression of genes (Tai et al. 2013), virus-mediated detailed connectivity (Fujiyama et al. 2011; Wall et al. 2013; Smith et al. 2016), and responses to channel-gating light with an illuminating apparatus placed in the brains of behaving animals (Valjent et al. 2009; Friedman et al. 2015; Smith et al. 2016; Stephenson-Jones et al. 2016; Bloem et al. 2017).

Recently, these particular circumstances have driven researchers to draw an extensive map of the striatum based on the specific connectivity between diverse neocortical areas and striatal subdomains (Hintiryan et al. 2016; Hunnicutt et al. 2016). Systematic tracer injections throughout the cortex have been combined with computer-aided analysis of big data, leading to demonstrations of objective delineation of numerous subdomains of the striatum. However, it remains to be clarified whether these corticostriatal projectomes are related to the internal structure-based map of the striatum that can be obtained by conventional histological techniques.

Aside from the issue of mapping, the striatum has a well-known, unique architecture called striosomes/matrix compartmentalization, which was first discovered two score years ago as heterogeneous mosaic patterns in acetylcholine histochemistry (Graybiel and Ragsdale 1978). Since then, numerous chemical markers have been used to visualize patch-like areas (striosomes) and the surrounding tissue (matrix). This contrasted profile has led to the dichotomy of classifying striatal tissue into the two compartments (Graybiel et al. 1981; Herkenham and Pert 1981; Gerfen 1984). One of the outstanding properties known for this compartmentalization is the selective innervation of dopamine neurons in the substantia nigra pars compacta (SNpc) only by the neurons residing in striosomes (Gerfen 1985; Fujiyama et al. 2011). The two compartments also differ in regulatory circuits by interneurons such as preferential localization of gap junction-coupled dendritic linkage in the matrix (Fukuda 2009). However, despite the usefulness of this dichotomy, it has been mentioned since the beginning of its discovery that not all striosomes are equal (Graybiel et al. 1981); specifically, they are extremely diverse in their immunoreactivities for various substances (Prensa et al. 1999). We focused on this complexity and have shown that the diverse compartmentalization depends on the position of the striosomes (Tajima and Fukuda 2013), which we hypothesize might suggest a possible clue to the divisions of the interior of the striatum.

In this study, we reconstructed three-dimensional distributions of diverse striosomes/matrix compartments from the complete serial sections across the entire striatum using immunohistochemistry for µ-opioid receptor (MOR), substance P (SP), and enkephalin (Enk). Importantly, these substances are key molecules in basal ganglia circuitry; specifically, axon terminals of striatonigral, direct pathway neurons and striatopallidal, indirect pathway neurons contain SP and Enk, respectively (Lee et al. 1997), while the endogenous ligand of MOR is Enk. The emergent structures represent novel intrastriatal domains that are occupied by different striosomal types. Moreover, the map based on these domains is well correlated to both the projection pattern from the neocortex and the uneven distributions of dopamine D1 receptor (D1R)- and D2 receptor (D2R)-expressing cells. The latter has special significance because D1R- and D2R-expressing cells correspond to striatonigral and striatopallidal neurons, respectively (Gerfen et al. 1990; Surmeier et al. 2007), and because there is a dominating idea that both populations are evenly distributed. The internal structure-based map of the striatum shown here will provide fundamental information for research in a variety of different fields, including analysis of animal models for many clinical disorders that affect the basal ganglia.

## Materials and methods

### Tissue preparation

All of experiments and animal procedures were performed according to the Guide for the Care and Use of Laboratory Animals (National institutes of Health Publications No. 80-23, revised 1986), and all of the protocols were approved by the Institutional Animal Care and Use Committees at Kumamoto University and Kurume University. All efforts were made to minimize the number of animals used and their suffering.

Six male C57BL/6J mice (21–26 g, 7–8 weeks old) and four male transgenic mice tagged for D_1_R-DARPP-32-Flag/D_2_R-DARPP-32-Myc (Bateup et al. 2008; 25–30 g, 8–10 weeks old) were used for histological analysis, and 37 male C57BL/6J mice were subjected to the tracer injection experiments.

For fixation of brains, animals were deeply anesthetized with sodium pentobarbital (100 mg/kg, intraperitoneal) and perfused via the ascending aorta with 0.01 M phosphate-buffered saline (PBS, pH 7.4) followed by 50 ml of 4% paraformaldehyde (PFA) in 0.1 M phosphate buffer (PB, pH 7.4) at room temperature. Brains were removed from the skull and stored overnight in the same fixative at 4 °C. The next day, the fixative was replaced by PBS containing 0.1% sodium azide.

### Injection of anterograde tracers

The anterograde tracers used were biotinylated 10 kDa dextran amine (BDA-10K, dissolved in 2.5% in saline, Molecular Probes) or phaseolus vulgaris-leucoagglutinin (Phal, 2.5% in 10 mM PB, pH 8.0; Vector Lab). BDA and Phal were injected stereotaxically into ten different cortical areas as shown in Table 1. Mice were anesthetized by inhalation of 0.5–2.0% isoflurane and mounted in a stereotaxic frame (SR-5M-HT, Narishige Scientific Instrument Lab). Then, a burr-hole was drilled in the appropriate position of the skull, and a glass microelectrode (outside tip diameter 40–50 µm) containing either of the two tracer solutions was stereotaxically inserted into the brain. BDA and Phal were injected iontophoretically into targeted sites by passing positive-pulsed 5–7 µA duty cycle (2 s on/2 s off) for 20 and 5 min, respectively. After surgery, mice were housed singly in small compartments that were temperature- (20 °C) and light-controlled (12 h light/12 h dark cycle). After a survival period of 7 days, the mice were perfusion-fixed as described above.

**Table 1:** Sites of tracer injections

Injection sites	Distance from the (bregma and midline)	Depth from the dura (mm)	Tracer
Primary motor area (M1)	(2.10 mm, 2.0 mm)	0.83	BDA
Primary somatosensory barrel area (S1BF)	(− 1.58 mm, 2.75 mm)	0.88	BDA
Primary visual area (V1)	(− 3.16 mm, 1.75 mm)	0.48	Phal
Primary auditory area (Au)	(− 2.92 mm, 3.75 mm)	0.63	Phal
Frontal association area (FrA)	(2.80 mm, 1.50 mm)	0.50	Phal
Agranular insular area (AI)	(1.54 mm, 2.75 mm)	2.25	Phal
Prelimbic area (PrL)	(1.98 mm, 0.25 mm)	1.20	Phal
Medial orbital area (MO)	(2.34 mm, 0.30 mm)	1.88	BDA
Cingulate area (Cg)	(0.98 mm, 0.25 mm)	1.0	Phal
Secondary motor area (M2)	(2.22 mm, 0.75 mm)	1.0	Phal

### Immunohistochemistry

Serial 50-µm-thick coronal sections were cut using a vibrating microtome (TTK-3000, Dosaka) from the brain block that contained the entire striatum. After cryo-protection in 25% sucrose in PBS, the sections were placed on aluminum foil and rapidly frozen in the vapor of liquid N_2_. The sections were then rapidly thawed in 25% sucrose in PBS, and then processed for triple-fluorescent immunohistochemistry, as previously described (Fukuda and Kosaka 2000; Miyamoto and Fukuda 2015) using slight modifications. The antibodies used are listed in Tables 2 and 3. Briefly, the sections were incubated with 5% normal donkey serum (Jackson ImmunoResearch) and 0.3% Triton-X in PBS overnight, with a mixture of guinea pig anti-MOR1 (1:5000, Millipore), rat anti-SP (1:500, Millipore, MAB356), and rabbit anti-Met-Enk (1:2500, Millipore, AB5026) antibodies for 7 days at 20 °C, with biotinylated donkey anti-rat IgG (1:250, Jackson ImmunoResearch, 712-065-150) overnight, and with a mixture of Cy3-conjugated donkey anti-guinea pig IgG (1:250, Millipore, AP193C), Alexa 647-conjugated donkey anti-rabbit IgG (1:250, Jackson ImmunoResearch, 711-605-152), and streptavidin-Alexa 488 (1:250, Molecular probes, S32354) overnight. The long incubation period with the primary antibodies was essential to improve permeation of the antibodies into the deep part of the sections and thus for confocal images of constant and sufficient quality throughout the depth of the sections (Fukuda et al. 1998).

**Table 2:** Primary antibodies used in this study

Antibody	Host species	Dilution	Source	cat. #/lot #
MOR1	Guinea pig	1:5000	Millipore	
SP	Rat	1:500	Millipore	MAB356/2857707
Met-Enk	Rabbit	1:2500	Millipore	AB5026/NMM1769933
CB D-28 k	Mouse	1:5000	Swant	300/07 (F)
Phal	Goat	1:5000	Vector Laboratories	AS-2224/X0228
Flag	Mouse	1:200	Sigma-Aldrich	F1804
c-Myc	Goat	1:2000	Novus biologicals	NB600-335

**Table 3:** Secondary antibodies used in this study

Antibody and fluorochrome	Conjugated fluorophore	Dilution	Source	code. #/lot. #
Donkey anti-rat IgG	Biotin	1:250	Jackson	712-065-150/95520
Donkey anti-guinea pig IgG	Cy3	1:250	Millipore	AP193C/2095331
Donkey anti-mouse IgG	Alexa 488	1:250	Jackson	715-545-151/130814
Donkey anti-rabbit IgG	Alexa 647	1:250	Jackson	711-605-152/107850
Donkey anti-goat IgG	Rhodamine red	1:500	Jackson	705-295-147/133388
Streptavidin	Alexa 488	1:250	Molecular probes	S32354
Streptavidin	Alexa 647	1:500	Jackson	016-600-084/131949

The second set of immunostaining was prepared from the animals injected with the tracers. Sections from Phal-injected animals were incubated in a mixture of goat anti-Phal (1:5000, Vector, AS-2224) and mouse anti-calbindin (CB; 1:5000, Swant, 300) antibodies and then in a mixture of Rhodamine red-conjugated donkey anti-goat IgG (1:500, Jackson, 705-295-147) and Alexa 488-conjugated donkey anti-mouse IgG (1:250, Jackson, 711-605-152). Sections from BDA-injected animals were incubated in mouse anti-CB antibody and then in a mixture of Alexa 488-conjugated donkey anti-mouse IgG and streptavidin-Alexa 647 (1:500, Jackson, 016-600-084). Note that BDA labeling in all figures are displayed with a pseudocolor of red as in color for Phal.

The third set of immunostaining was prepared for sections from the transgenic mice. To investigate the distributions of D1R- and D2R-positive cells within the diverse compartments of the striatum, three sets of triple-immunostaining were performed in 4 adjacent sections using a mixture of mouse anti-Flag (1:200, Sigma, F1804) and goat anti-c-Myc (1:2000, Novus biologicals, NB600-335) antibodies combined with one of the following antibodies: guinea pig anti-MOR1, rat anti-SP, and rabbit anti-Met-Enk antibodies. The sections were further incubated with the secondary antibodies listed in Table 3.

Immunostained sections were mounted in Vectashield (Vector Laboratories) and examined using a confocal laser-scanning light microscope (C2, Nikon), which was equipped with three single laser beams, 488, 543, and 633 nm in wavelength, and a filter set of BA 515/30, BA 590/50, and 650 LP. Control sections were prepared by omission of primary antibodies and by mismatching secondary antibodies; both provided only weak nonspecific staining.

### Confocal laser scanning microscopy

Images for confocal laser scanning light microscopy (CLSM) were obtained using the × 4 (Plan Apo, N.A. = 0.2, Nikon) and × 40 (Plan Fluor, N.A. = 0.75, Nikon) objective. The × 4 objective was used to visualize the whole striatum in a single frame in CLSM, whereas the × 40 objective was used to identify and analyze striatal neurons and tracer-labeled axon terminals of cortical afferents. The size of each frame was 1024 × 1024 pixels, and the intensity of the signal in each pixel was recorded at 8-bits for each channel. All of the image data from the same animal were obtained by maintaining the CLSM settings constant, such as the laser power and the gains of the photomultipliers. Images of the optical slices were acquired from the section surface to the bottom at the preset optimal step size and were stored as a stacked file for each frame using the three single laser beams alternately at each z-position of the stage to collect images for the different fluorescence signals.

### Analysis

To determine the rostrocaudal extent of the striatum, the CLSM images were acquired with a × 4 objective from the serial coronal sections containing the whole striatum in animals, and the sections were triple-labeled for MOR, SP, and Met-Enk. The distance of each section from the reference section that contained the center of the anterior commissure crossing the midline was estimated by multiplying the section thickness (50 µm) by the number of intervening sections.

Striosomes were defined in MOR-, SP-, and Enk-immunohistochemistry as patchy domains that showed higher immunoreactivity than the surrounding tissue (matrix), with a clear boundary and with morphological features common to the previous observations: they had round, oval or elongated shapes with an occasional bifurcation or intervening thin bridges and had a relatively constant transverse diameter (Tajima and Fukuda 2013). Striosomes were selected objectively in the following procedure using the public domain program ImageJ (v.1.47) and the application Neurolucida (Fig. S1 in the Supplementary material): (1) the original images in single channels were acquired by CLSM; (2) after inversion of gray levels in each image (no signal = 0, maximum level = 255), background signals were subtracted via the “Rolling Ball” command in ImageJ; (3) images were thresholded at a constant gray level for each channel to obtain binary images; (4) noise was reduced using median filter processing; (5) gray levels were inverted again; (6) white color in the binary images was replaced by red color; (7) the extracted pixels shown in red were applied to the original images; and (8) each striosome was traced using Neurolucida. After these procedures, several weakly stained, but clearly discernible striosomes remained. This was because the intensity of labeling in both striosomes and the matrix was diverse depending of their positions inside the striatum even in a single section (Graybiel et al. 1981; Prensa et al. 1999); thus we found it was necessary to rescue such weakly stained striosomes by the visual inspection, which had an advantage over extracting striosomes through automatic-only or inspection-only procedures.

The numbers of D1R- and D2R-expressing neurons were counted in a set of four serial, triple-stained sections, each of which was labeled for the D1R, D2R, and one for the MOR, SP, and Enk; specifically, MOR was labeled in the first and fourth sections, SP was labeled in the second section, and Enk was labeled in the third section. The types of striosomes were evaluated by comparing their immunoreactivities for the three substances in the neighboring sections. Contours of the individual striosomes were traced and applied to the sections for neuron counting using Neurolucida. Cells were counted by a bias-free method disector (Sterio 1984; Miyamoto and Fukuda 2015).

### Statistics

The proportions of D1R- and D2R-expressing neurons were compared among the compartments using the Tukey–Kramer test with *p* < 0.05 considered statistically significant.

## Results

### Immunohistochemical diversity in striosomes

**Fig. 1:**
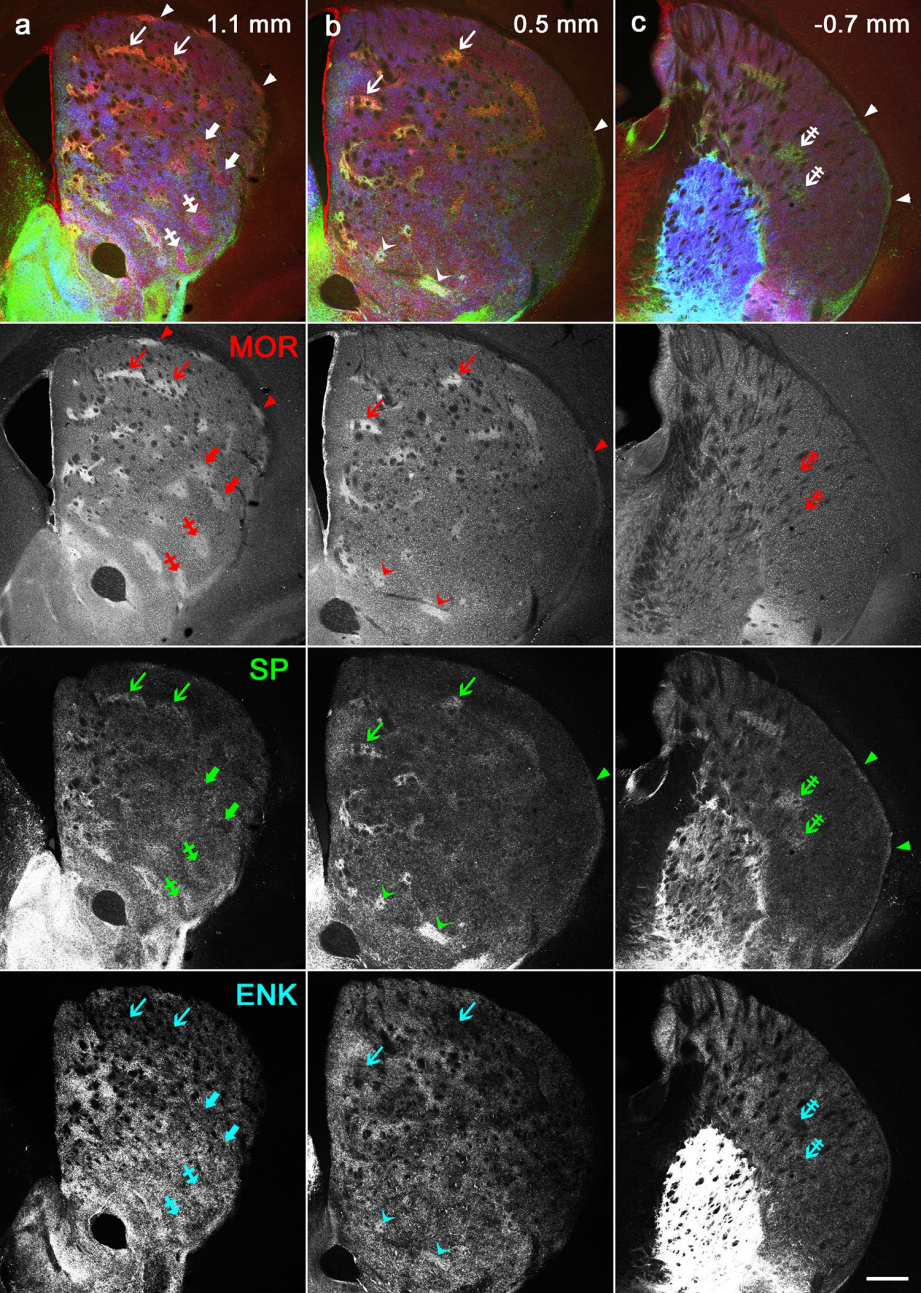
Diverse immunoreactivities in striosomes/matrix compartmentalization. The pseudo-color images in **a, b**, and **c** consist of MOR (red), SP (green) and Enk (blue) immunoreactivities, which are presented separately in the lower panels. The position along the rostrocaudal axis is shown as the distance from the level of the anterior commissure crossing the midline that is positioned 0.14 mm rostral to the bregma. Striosomes labeled for both MOR and SP are indicated by thin arrows, those for MOR only and SP only are indicated by thick arrows and double-crossed arrows, respectively, those for both MOR and Enk are indicated by crossed arrows, and those for all of MOR, SP, and Enk are indicated by thin arrowheads. Thick arrowheads indicate subcallosal streaks that also show diverse immunoreactivities for MOR only (**a**), MOR and SP (**b**), and SP only (**c**). Scale bar = 300 µm

Coronal sections containing the entire striatum were cut serially and processed for triple immunohistochemistry using antibodies against MOR, SP, and Enk (Fig. 1). The rostrocaudal extent of the mouse striatum was between 1550 µm rostral to the anterior commissure (AC; + 1550 µm) and 2150 µm caudal to the AC (− 2150 µm). Both SP- and Enk-immunoreactivities were observed in axon terminals, whereas somata were devoid of immunoreactivities for both. This is because labeling of somata requires antibodies for the precursor forms, that is, preprotachykinin for SP and preproenkephalin for Enk (Lee et al. 1997). MOR-immunoreactivity was observed as diffuse amorphous staining in neuropils, possibly reflecting the presence of receptors on fine dendritic segments.

We first confirmed our previous observations (Tajima and Fukuda 2013) that striosomes showed various immunoreactive patterns in response to the three immunohistochemical markers (Fig. 1). Then we compared the combination of these markers in all the striosomes contained in serial sections through the whole striatum and found that striosomes could be classified into five types: those detectable only by MOR (MOR-only striosomes) or SP (SP-only striosomes), those detectable by both MOR and SP (MOR/SP striosomes) or by both MOR and Enk (MOR/Enk striosomes), and those detectable by MOR, SP, and Enk (MOR/SP/Enk striosomes). SP- and Enk-double positive striosome lacking MOR labeling was not encountered. Both SP- and Enk-immunoreactive axon terminals in striosomes are thought to originate from neurons in the same striosome, which is based on the ground that striosomal neurons give off local collateral axons exclusively inside their home striosome, whereas axon collaterals of matrix neurons do not enter striosomes (Penny et al. 1988; Kawaguchi et al. 1989; Fujiyama et al. 2011). Lack of direct monosynaptic connections between striosomes and matrix has also been confirmed physiologically (Lopez-Huerta et al. 2016).

**Fig. 2:**
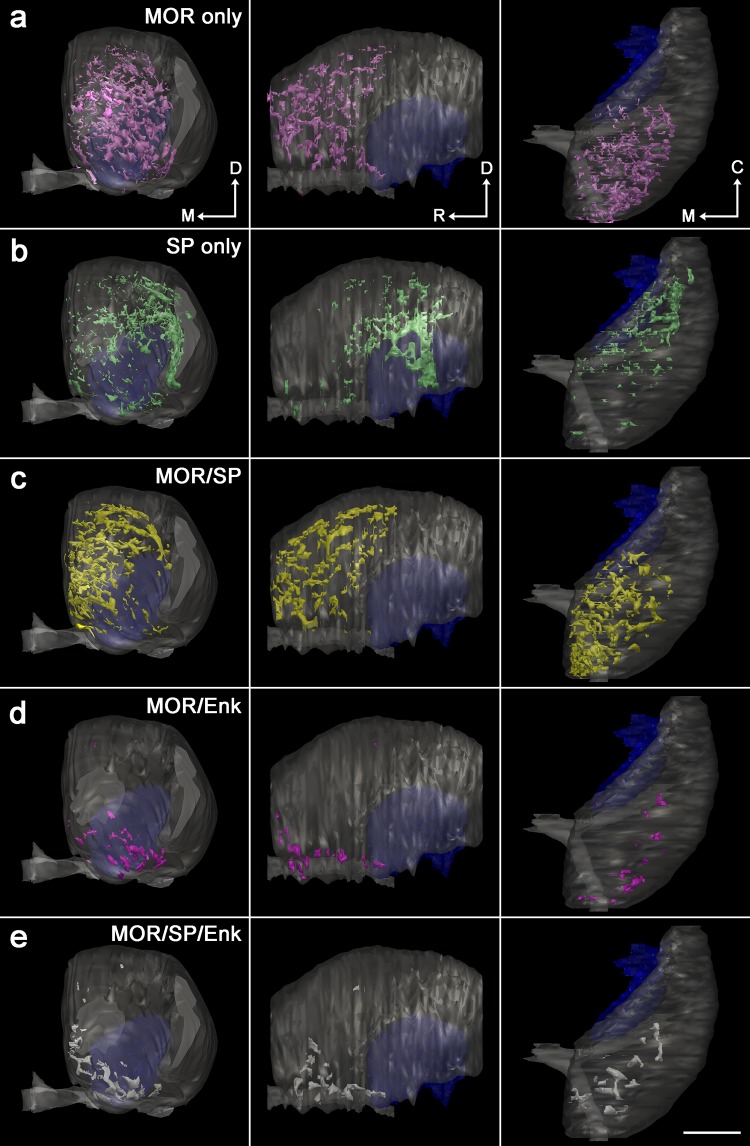
Five types of striosomes occupying particular spatial domains inside the striatum. Three-dimensional reconstructions from the complete serial sections are shown in front (left), lateral (middle), and top (right) views. MOR-only (**a**) and MOR/SP (**c**) striosomes are located predominantly in the rostral half of the striatum, whereas SP-only striosomes (**b**) are located in the caudal half. MOR/Enk (**d**) and MOR/SP/Enk (**e**) striosomes are distributed in the most ventral position close to the nucleus accumbens. The globus pallidus is shown in dark-blue. *M* medial, *D* dorsal, *R* rostral, *C* caudal. Scale bar = 1 mm

**Fig. 3:**
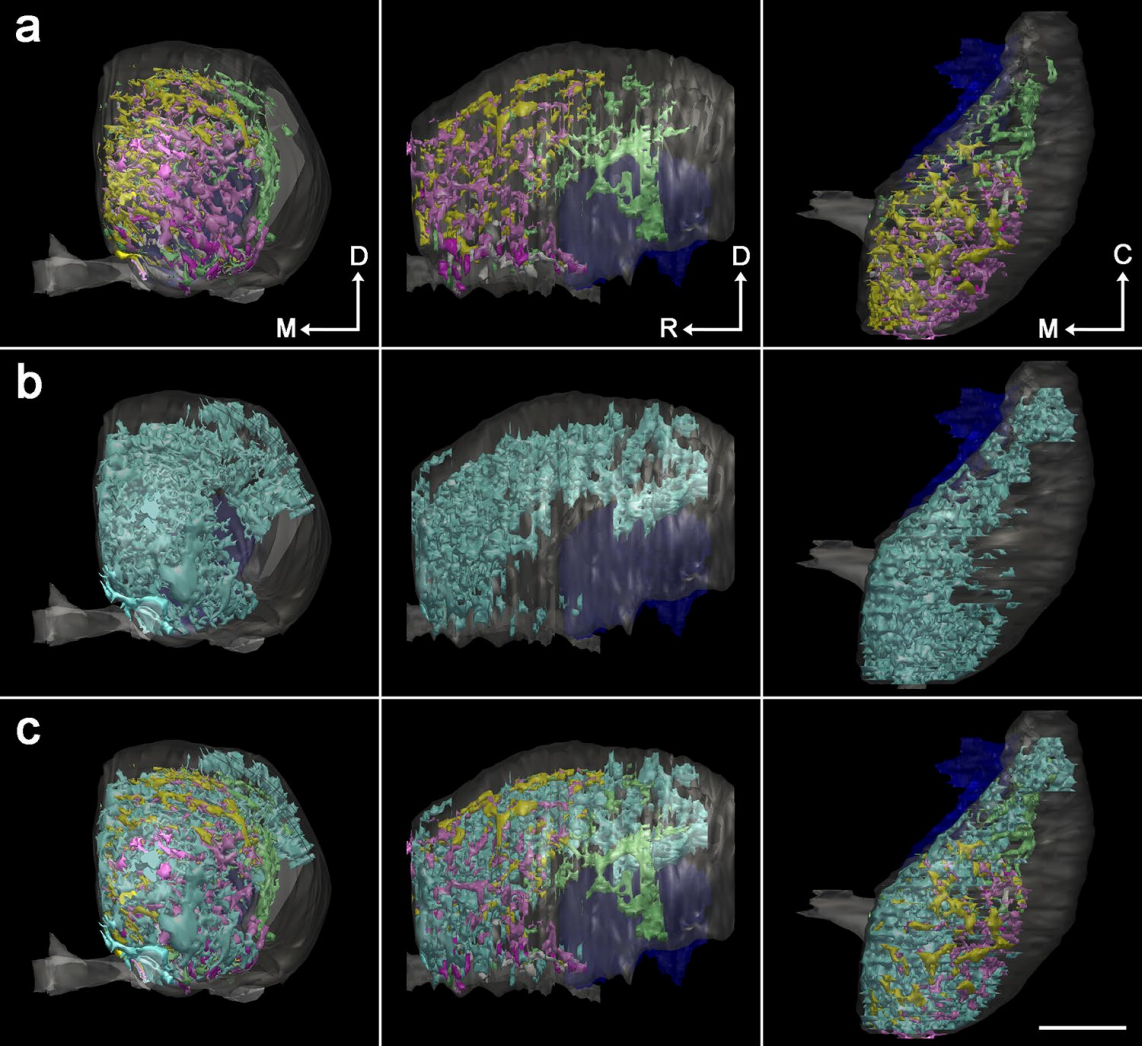
The striosome-free space surrounding the striosome-rich part. Frontal, lateral, and top views are shown in the left, middle, and right panels, respectively. **a** Combined image of the five types of striosomes shown by the same colors that are used in Fig. 2. Both MOR/SP striosomes (yellow) and MOR-only striosomes (pink) are distributed mainly in the rostral half of the striatum with the former taking a more medial position. Note the striosome-free space in the dorsolateral position surrounding the accumulation of striosomes. **b** Three-dimensional distribution of Enk-rich islands identified in the matrix. **c** Combined image of striosomes and Enk-rich islands showing interdigitation of both compartments. Matriceal Enk-rich islands do not invade the striosome-free space. Scale bar = 1 mm

As a next step to explore the three-dimensional distribution of the newly defined five types of striosomes, individual striosomes in each section were selected by sequential procedures consisting of digitization, thresholding for binary images, and tracing using a computer-assisted neuron tracing system (Figure S1 in the Supplementary Material). Then, we reconstructed the five types from complete serial sections covering the entire striatum (Fig. 2), leading to the finding that each type had its own domain inside the striatum. MOR-only striosomes were located in the rostral half of the striatum (Fig. 2a), whereas SP-only striosomes were found mainly in the caudal half (Fig. 2b). MOR/SP striosomes had a distribution similar to and intermingled with MOR-only striosomes (Fig. 2c), but as a population, MOR/SP striosomes were located slightly more medially to MOR-only striosomes (Fig. 3a). In contrast with these three types that occupied the central part of the striatum, both MOR/Enk and MOR/SP/Enk striosomes were detectable in the ventromedial, peripheral part close to the nucleus accumbens (Fig. 2d, e). The specific distribution pattern of each striosomal type shown in Fig. 2 was consistent with our previous qualitative observations (Tajima and Fukuda 2013) and was further confirmed by quantitative analyses performed in three animals (Figures S2 and S3 in the Supplementary Material). Quantification of the area of striosomes in three mice resulted in the following proportions: MOR-only striosomes, 28.2 ± 2.3% (mean ± SD); SP-only striosomes, 19.6 ± 2.9%; MOR/SP striosomes, 33.3 ± 2.7%; MOR/Enk striosomes, 6.3 ± 1.5%; MOR/SP/Enk striosomes, 12.7 ± 2.7%. For each striosomal type, the proportion of striosomal areas located rostral to the anterior commissure crossing the midline was 90.4 ± 7.0% in MOR-only, 8.1 ± 2.8% in SP-only, 76.6 ± 4.4% in MOR/SP, 92.2 ± 9.3% in MOR/Enk, and 68.5 ± 8.9% in MOR/SP/Enk striosomes.

There has been accumulating evidence that striosomes that appear as patchy profiles in individual sections actually form a continuous labyrinth in many species, including the cat (Groves et al. 1988), rat (Desban et al. 1993), mouse (Breuer et al. 2005), monkey (Mikula et al. 2009), and human (Manley et al. 1994). The present analysis confirmed this, and further revealed that the five types of striosomes formed a single labyrinth. Double-positive striosomes were connected to single-positive striosomes in such a manner that each of the two immunoreactivities in double-positive striosomes gradually decreased when moving toward a single-positive striosome that was detectable with the other marker.

**Fig. 4:**
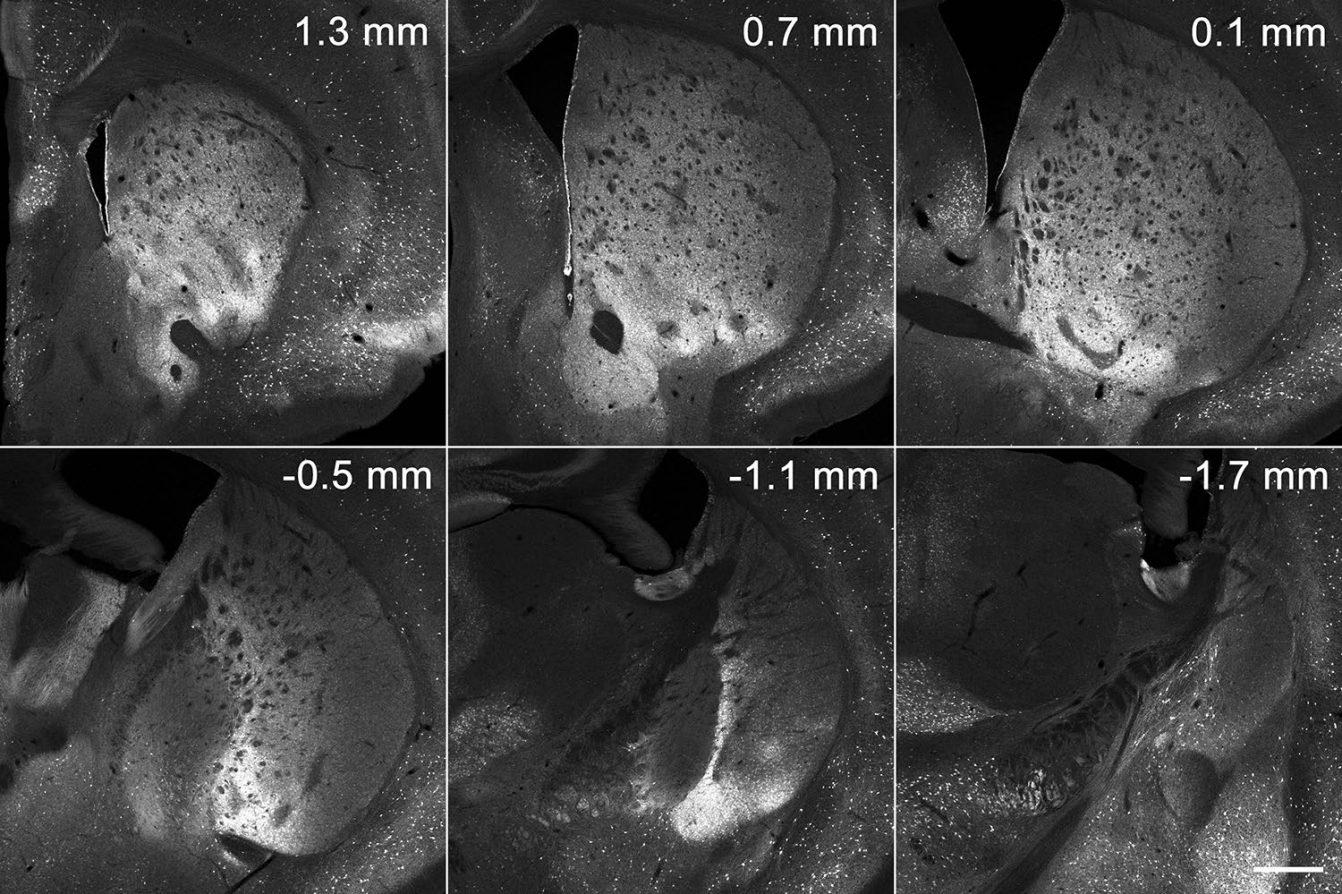
Immunoreactivity for CB. Striosomes are detectable as poorly labeled patchy areas surrounded by the CB-immunoreactive matrix. Dorsolateral parts at positions of 0.7 mm and caudally are devoid of striosomes and show relatively weak CB-immunoreactivity compared to the matrix. Also note the intense CB labeling in the ventromedial part of the striatum. Scale bar = 0.5 mm

**Fig. 5:**
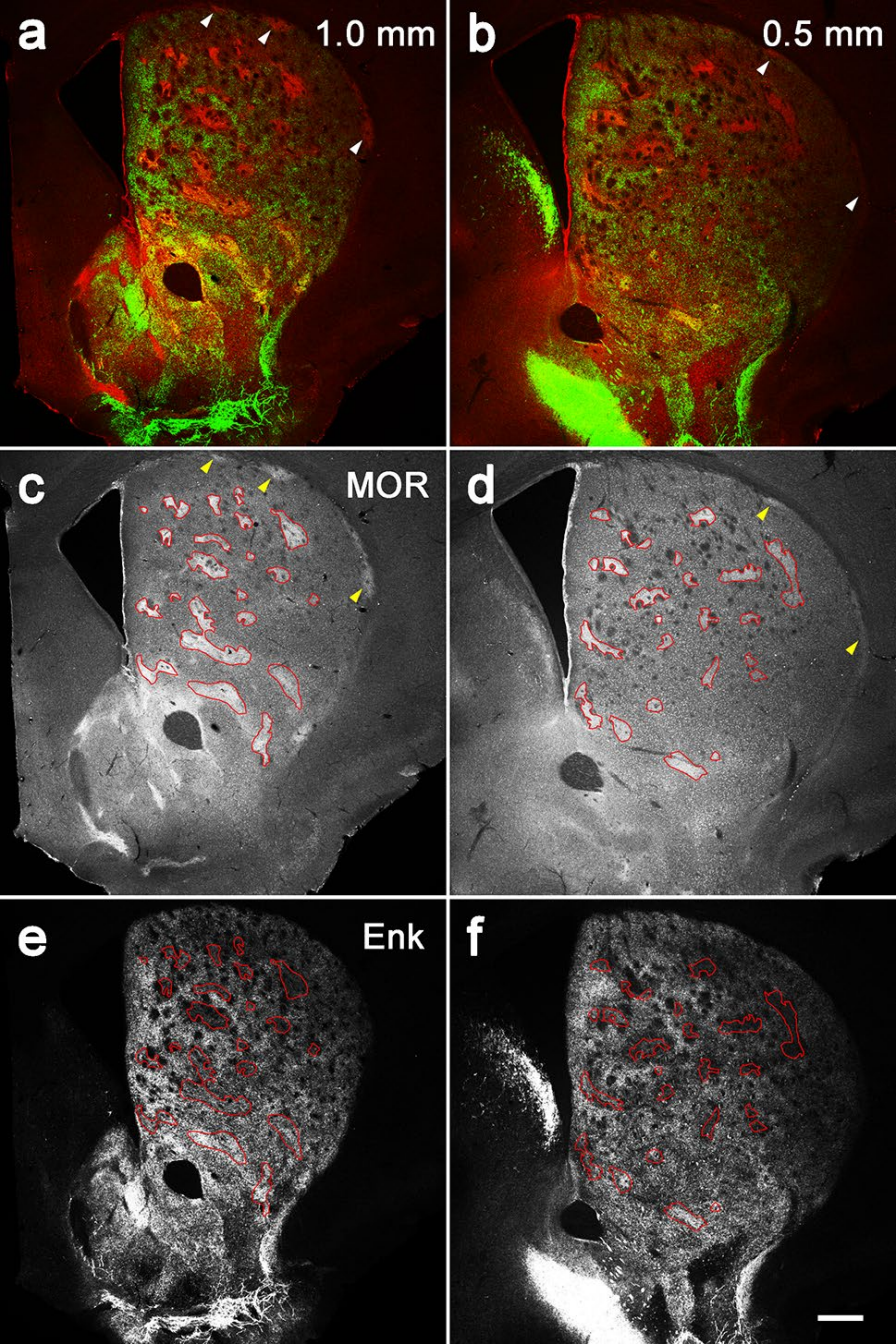
Enk-rich islands in the matrix. **a, b** Double immunohistochemistry for MOR (red) and Enk (green) at two different positions along the rostrocaudal axis. Both MOR and Enk immunoreactivities are seen as patchy structures but they do not overlap with each other in most areas except for the ventromedial part. Arrowheads indicate subcallosal streaks. **c, d** Tracing of MOR-positive striosomes. **e, f** Superimposition of the contours in **c, d** onto the images of Enk labeling. It is evident that Enk-rich islands are located not in striosomes but in the matrix except for the ventromedial position. MOR-positive striosomes are located in the regions where Enk-immunoreactivity is weak, especially in the dorsal positions. Scale bar = 300 µm

One remarkable finding obtained from the superimposition of all reconstructions from the five types is the existence of a striosome-free space that occupied a large volume in the dorsolateral part of the striatum (Fig. 3a). As a traditional view, this striosome-free space might be interpreted as a large expansion of matrix into the lateral part of the striatum. However, the striosome-free space could be discriminated from the matrix on two grounds. First, immunohistochemistry for calbindin (CB), a general marker of the matrix (Gerfen et al. 1985; Kawaguchi et al. 1989; Liu and Graybiel 1992) that surrounds CB-poor striosomes (Fig. 4), resulted in very weak staining in the dorsolateral domain that corresponded to the striosome-free space. Second, the striosome-free space lacked a novel inhomogeneous pattern of Enk-immunoreactivity that was detectable in the matrix (Figs. 3b, 5). The single channel-confocal image for Enk represented island-like profiles that resembled striosomes with similar sizes. However, these Enk-rich islands resided not in striosomes but in the matrix (Tajima and Fukuda 2013), leading to an interdigitating pattern of Enk-rich islands and striosomes (Fig. 3c). These Enk-rich islands are thought to correspond, at least partially, to matrisomes that have been visualized in diverse experimental conditions (Flaherty and Graybiel 1993; Eblen and Graybiel 1995; Tai et al. 2013). The superimposition of Enk-rich islands and striosomes clearly indicates the existence of the striosome-free space (Fig. 3c), which was not a lateral expansion of CB-rich matrix.

The dorsolateral position of the striosome-free space might lead to confusion with another peculiar structure called subcallosal streak (Fig. 1, Figure S4a–c in the Supplementary material) that has been repeatedly described in rodent striatum as narrow areas showing striosome-like immunoreactivities just beneath the corpus callosum and the external capsule (Desban et al. 1993; Tokuno et al. 1996; Prensa and Parent 2001; Breuer et al. 2005). As were the case with striosomes, subcallosal streaks also showed diverse immunoreactivities (Fig. 1, Figure S4 in the Supplementary material). Three-dimensional reconstruction revealed that subcallosal streaks were connected to striosomes but that this was only at the rostral tip of the striatum; more caudally throughout the striatum, there was no connection between the two structures. Therefore, the striosome-free space intervened and occupied a large area between a mass of striosomes and the subcallosal streaks (Figure S4d-f in the Supplementary material).

**Fig. 6:**
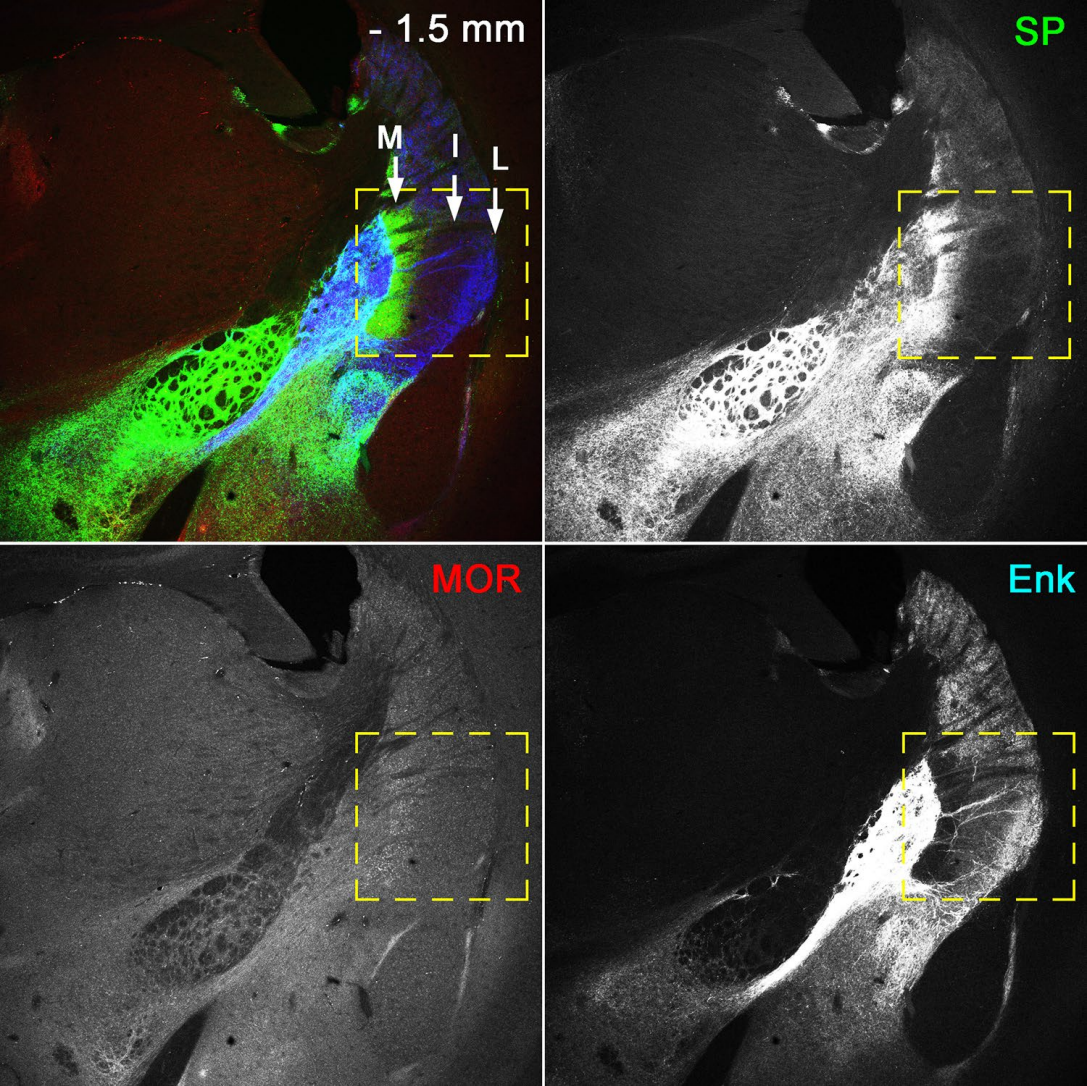
The tri-laminar part at the most caudal striatum. The pseudo-color image consists of SP (green)-, MOR (red)-, and Enk (blue)-immunoreactivities, which are also presented separately. The rectangle indicates the tri-laminar part, where the medial band (M) is characterized by intense SP-/almost no Enk-labeling, the intermediate band (I) is characterized by almost no SP-/weak Enk-labeling, and the lateral band (L) is characterized by almost no SP-/intense Enk-labeling. Scale bar = 0.3 mm

Close observations at different rostrocaudal positions revealed additional features in labeling patterns. The first one was observed as a domain of intense CB labeling in the ventromedial part at the mid-rostrocaudal level (Fig. 4). This domain contained MOR/SP/Enk and MOR/Enk striosomes (Fig. 2d, e). Another characteristic domain was located at the most caudal part and consisted of tri-laminar bands just lateral to the globus pallidus (Fig. 6), where the most medial band was characterized by highly intense labeling for SP and the absence of Enk labeling, the most lateral band exhibited intense Enk labeling and the absence of SP labeling, and the intermediate band had weak Enk labeling and lacked SP labeling.

**Fig. 7:**
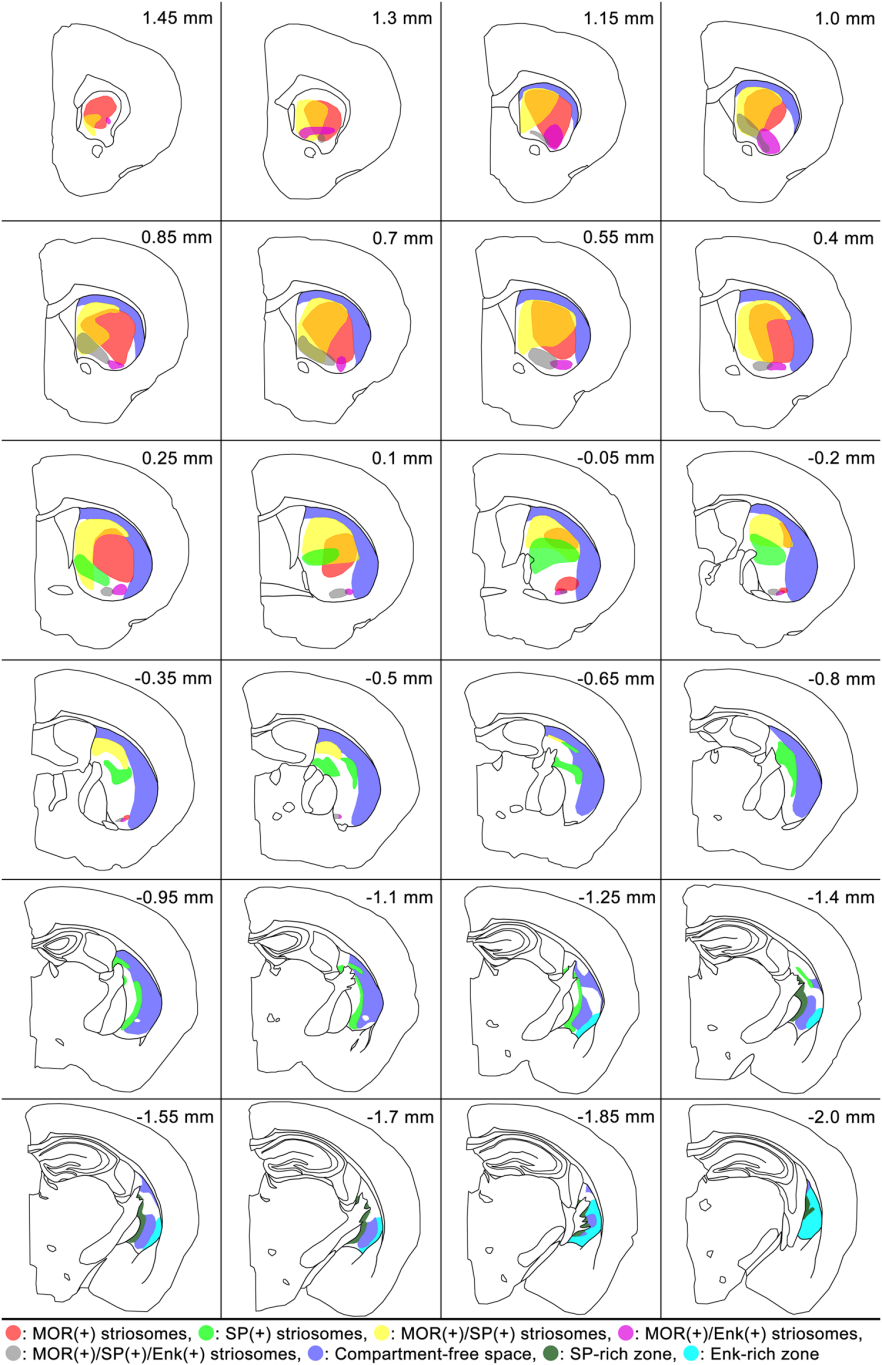
Distributions of the five types of striosomes and striosome-free space. Areas where different types overlap are shown in intermediate colors. The striosome-free space (light purple) is located in the dorsolateral part of the striatum. White areas belong to the CB-positive matrix where striosomes are relatively sparse. The SP-rich zone and Enk-rich zone in the most caudal, tri-laminar part are shown in different colors. The position along the rostrocaudal axis is shown as the distance from the level of the anterior commissure crossing the midline that is positioned 0.14 mm rostral to the bregma

Overall, the striatum could be divided into three major parts, (1) the striosome-rich part, (2) striosome-free space, and (3) the most caudal tri-laminar part. The striosome-rich part contained five types of striosomes, each occupying a particular domain. According to these classifications, we drew an internal structure-based map of the striatum along the entire rostrocaudal axis of the striatum (Fig. 7).

**Fig. 8:**
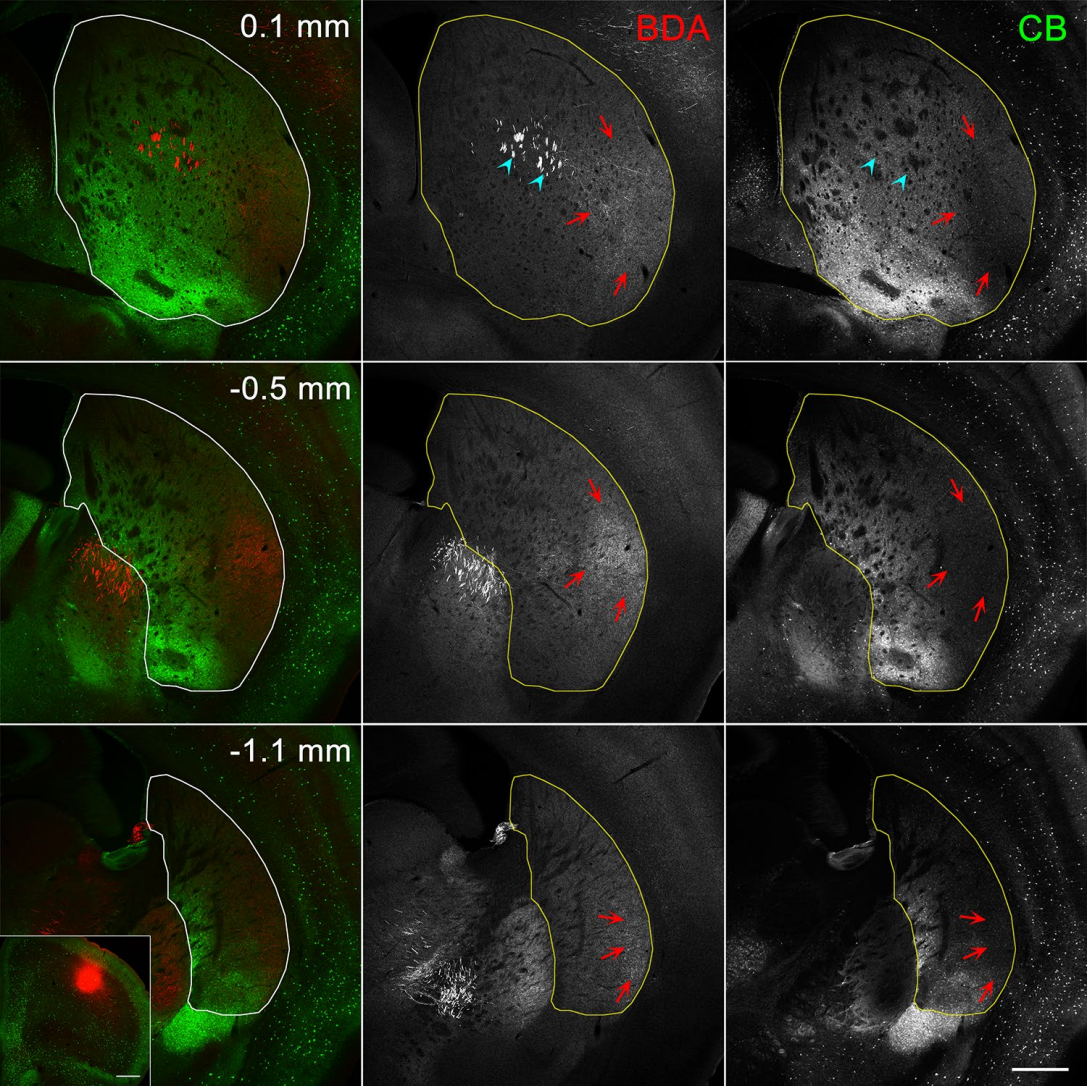
Innervation of striosome-free space by cortical afferents from the M1. The site of BDA injection is shown in the inset. BDA-labeled axons and axon terminals (arrows) are located predominantly in the lateral, CB-poor/striosome-free space. Arrowheads indicate labeling of descending axons in the white matter. Scale bars = 0.5 mm

As a next step, we determined that this map was in good correspondence to the distribution of afferent axons from diverse sources in the cerebral cortex. First, and most characteristically, axons labeled by anterograde tracers injected into the primary motor area were distributed predominantly in the striosome-free space (Figs. 8, 10b–e). Moreover, axons from the primary somatosensory area also targeted the striosome-free space (Fig. 10b–e), with the topography of somatosensory afferents located dorsal to motor afferents. Much fewer axons originating from these two areas innervated the striosome-rich part near the striosome-free space, where they still avoided striosomes. Further analysis of afferents from both the primary auditory (Fig. S5 in the Supplementary material) and primary visual areas also supported the general principle that axons from the primary motor and sensory areas selectively target the striosome-free space with topography inside the space (Fig. 10b–f).

**Fig. 9:**
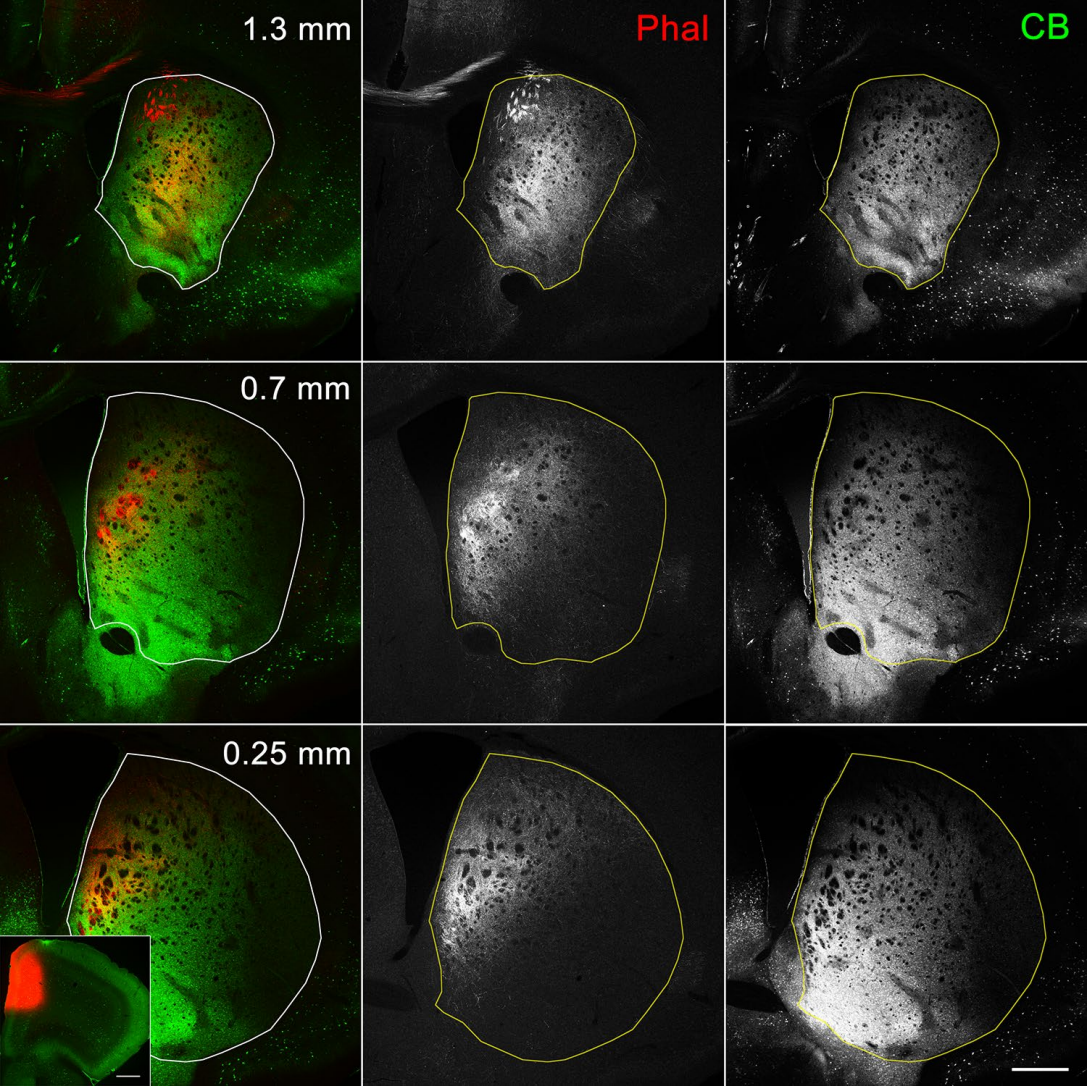
Innervation of the striosome-rich region by afferents from the prelimbic area. Labeled axons and axon terminals are distributed in the dorsomedial, striosome-rich region that overlaps the domain of MOR/SP striosomes. Scale bars = 0.5 mm

**Fig. 10:**
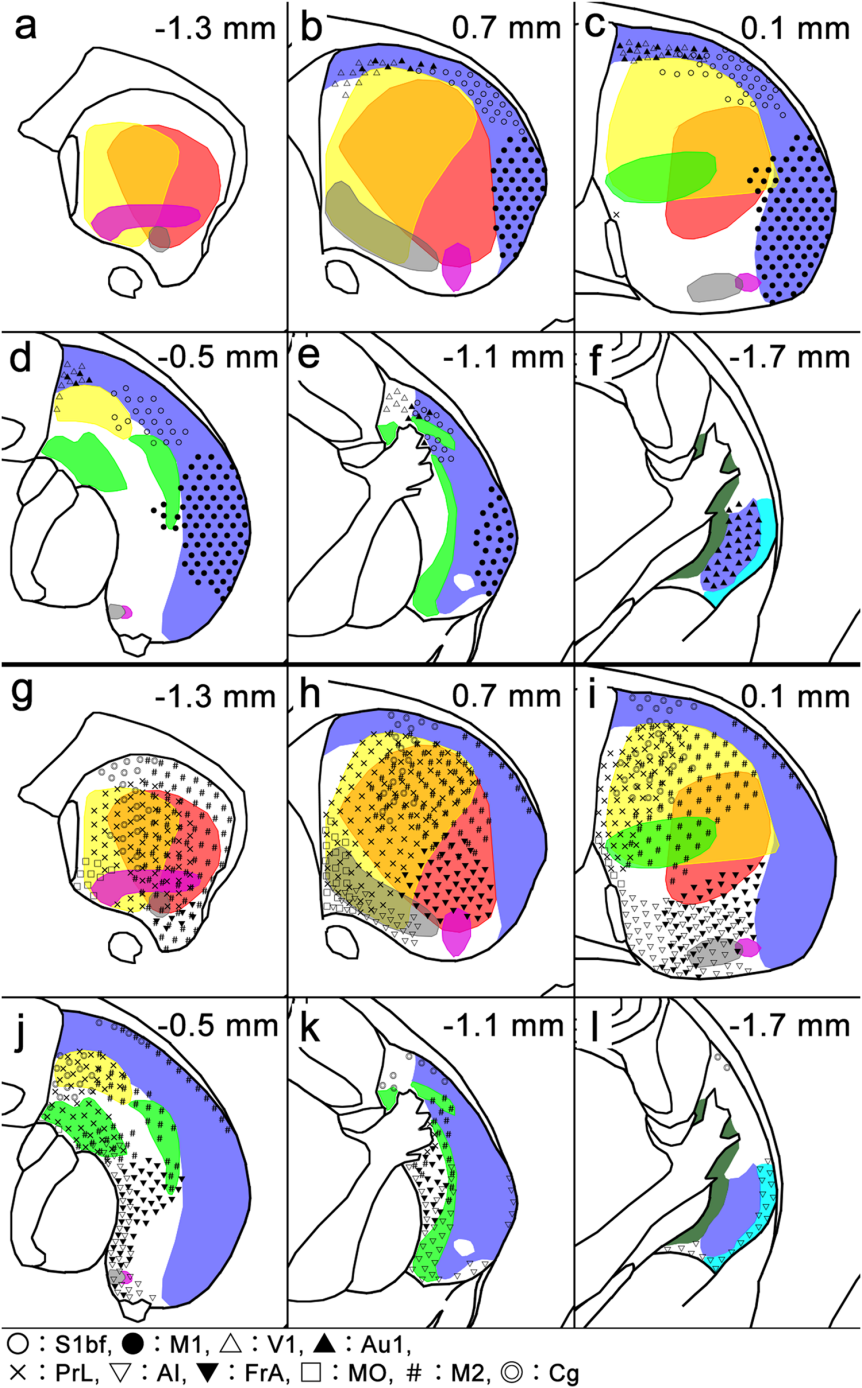
Contrasting projection patterns of corticostriatal axons between the striosome-free space and the striosome-rich region. Domains of various compartmentalization are shown by the same colors that are used in Fig. 7. **a**–**f** Anterograde tracer-labeled axon terminals originating from the primary motor (M1), somatosensory barrel (S1bf), visual (V1), and auditory (Au1) cortices are distributed predominantly in the striosome-free space (light purple) with topography for individual cortical origins. **g**–**l** Axon terminals from the prelimbic (PrL), agranular insular (AI), frontal association (FrA), medial orbital (MO), anterior cingulate (Cg), and secondary motor (M2) cortices are distributed in the striosome-rich region with the following topographical patterns: PrL axons are located in the dorsomedial region that corresponds to MOR/SP domain (**g**–**j**), AI axons are located in the ventral region, including MOR/SP/Enk domain (**h**–**j**) and the lateral part of the most caudal tri-laminar bands (**l**), FrA axons are located in the ventrolateral (MOR-only domain in **h**) and ventral (uncolored, CB-positive matriceal domain in **i**–**k**) regions, MO axons are located in the ventromedial peripheral region (**g**–**i**), M2 axons are located in the dorsolateral region consisting of MOR/SP, MOR-only, and SP-only domains (**g**–**k**), and Cg axons are located in the medial and mid-mediolateral regions that overlap with PrL projection areas (**g**–**j**). Axons originating from the M2 and Cg are also found in the subcallosal streaks (**h**–**l**)

This remarkable projection pattern was in sharp contrast to the distribution of afferents from the associational/limbic cortical areas. Axons from the prelimbic area targeted the medial region of the striosome-rich part in the rostral and middle striatum (Figs. 9, 10g–k), distributing in both striosomes and matrix while avoiding the striosome-free space. The target area of prelimbic axons corresponded to the MOR/SP domain. Similarly, axons originating from the frontal association area (FrA) targeted the striosome-rich part, mainly in the matrix, broadly along the rostrocaudal axis (Fig. 10g–k, see also Figure S6 in the Supplementary material). FrA axons were distributed in the ventrolateral region corresponding to the MOR-only domain rostrally (Fig. 10h) and in the ventral region corresponding to the CB-positive matriceal domain more caudally (Fig. 10i–k).

Axons from the secondary motor (M2) area were distributed in the subregion of the striosome-rich part that has been so far regarded as the “dorsolateral sensorimotor part” (Fig. 10g–k), but most of the M2 afferents avoided the more lateral, striosome-free space. Axons from M2 in the striosome-rich part were detectable in both striosomes and the matrix. The types of striosomes innervated by M2 were MOR/SP-, MOR-only, and SP-only.

Projections from the anterior cingulate cortex targeted the dorsomedial region of the striosome-rich part (Fig. 10g–l). These axons resided in the MOR/SP domain, overlapping with prelimbic axons, but anterior cingulate axons avoided the striosomes. Some cingulate axons were also found in the most dorsal region of the rostral striosome-free space.

Axons form the medial orbital area distributed in the most ventromedial region of the striosome-rich part (Fig. 10g–i), where they targeted striosomes that were identified as MOR/SP striosomes. Distribution of axons originating from the agranular insula showed a unique pattern, as they targeted the most ventral striatum mainly in the CB-intense region (Fig. 10h–l, see also Figure S7 in the Supplementary material) and entered MOR/SP/Enk positive striosomes. Axons from the agranular insula also innervated the most caudal tri-laminar part, where they selectively distributed in the most lateral, Enk-rich band (Fig. 10l, see also Figure S7 in the Supplementary material, − 1.4 mm).

The present results show good topographical correspondence between the corticostriatal projections and the striatal division: the primary motor and sensory areas innervated the striosome-free space, whereas the associational/limbic areas targeted the striosome-rich part of the striatum. Further, the topography could be subdivided within both the striosome-free space and striosome-rich part as summarized in Fig. 10.

**Fig. 11:**
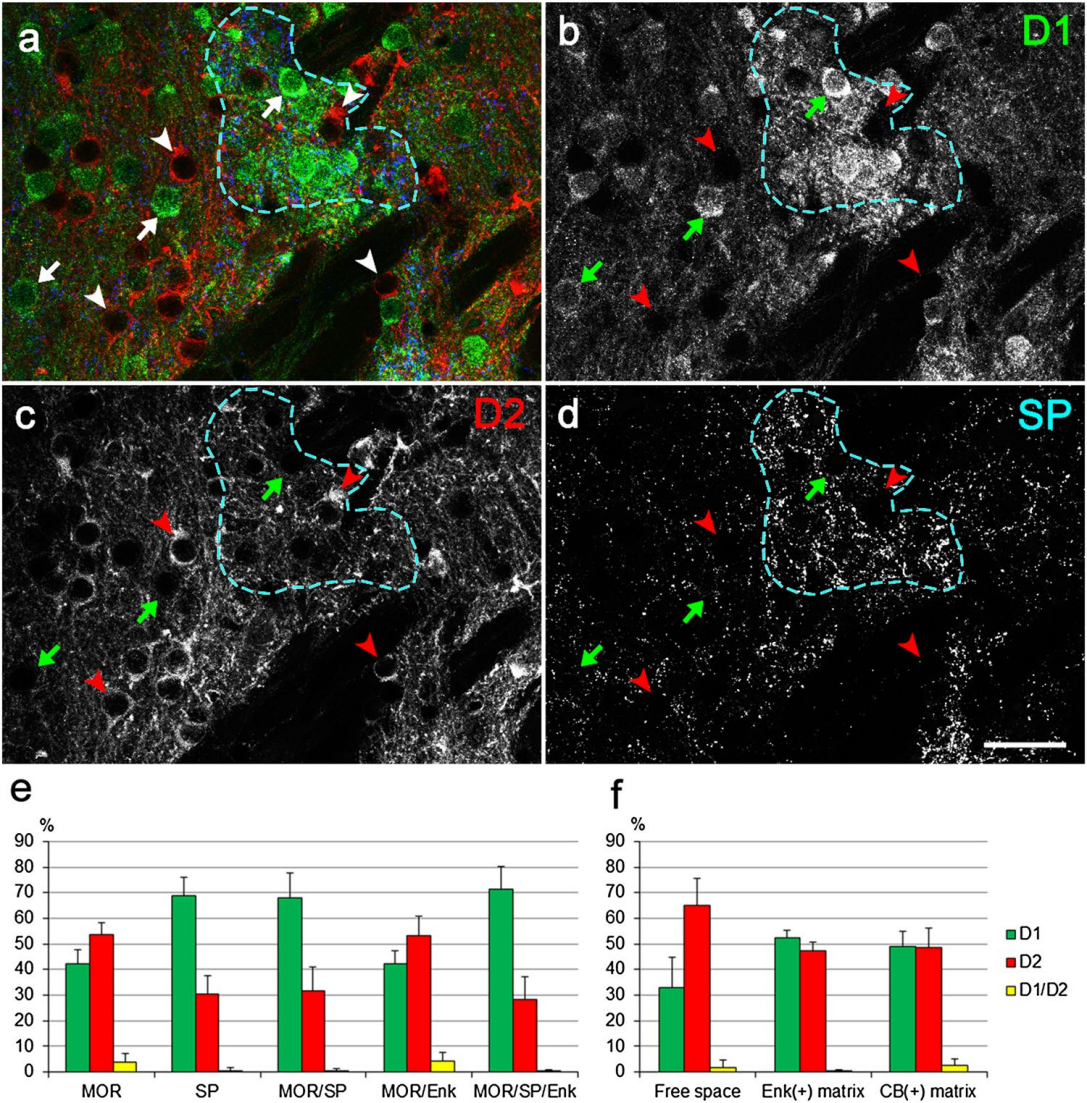
Analysis of the distributions of D1R- and D2R-expressing cells. **a** Triple labeling consists of D1R- (green), D2R- (red) and SP- (blue) immunoreactivities, which are shown separately in **b**–**d**. Arrows and arrowheads indicate D1R- and D2R-expressing cells, respectively. The contour of a striosome is shown by blue dotted line. **e, f** Uneven distributions of D1R- and D2R-expressing neurons depending on the striatal compartments. The proportions of D1R- (green), D2R- (red), and D1R and D2R-double expressing (yellow) neurons in diverse compartments are shown as the mean ± SD. **e** The five types of striosomes. **f** Striosome-free space and two matriceal regions. Three types of SP-containing striosomes (SP, MOR/SP, MOR/SP/Enk) show D1R ratios of approximately 70% that are significantly higher than those of the other compartments. Alternatively, the striosome-free space shows a D2R ratio of 64% that is significantly higher than all other compartments (Tukey–Kramer, *p* < 0.05). Details of statistical analysis are provided in Figure S8 in the Supplementary material. Scale bar = 30 µm

Finally, we further assessed the validity of the internal structure-based map of the striatum shown here in terms of its relationship to the composition of dopamine D1R- and D2R-expressing neurons, which is another key to understand striatal organization, using D1-DARPP-32-Flag/D2-DARPP-32-Myc transgenic mice (Bateup et al. 2008). In spite of a general belief that these two populations are almost in equal number, our quantitative analysis of 6894 neurons from three animals has revealed that the proportion changes considerably depending on the types of compartments (Fig. 11, see also Figure S8 in the Supplementary material). The proportion of D1R-expressing neurons in MOR/SP, SP-only, and MOR/SP/Enk striosomes was 67.9 ± 9.7% (mean ± SD; *n* = 22 striosomes, 1667 cells), 69.0 ± 6.9% (*n* = 12 striosomes, 753 cells), 71.6 ± 8.6% (*n* = 15 striosomes, 1345 cells), respectively; whereas that in MOR-only and MOR/Enk striosomes was 42.3 ± 5.3% (*n* = 12 striosomes, 956 cells) and 42.3 ± 5.1% (*n* = 10 striosomes, 573 cells), respectively. On the other hand, the proportion of D1R-expressing cells in the striosome-free space, Enk-positive islands in the matrix, and CB-intense matrix was 33.2 ± 11.8% (*n* = 11 positions, 601 cells), 52.2 ± 3.2% (*n* = 11 islands, 621 cells), and 48.9 ± 6.2% (*n* = 11 positions, 378 cells), respectively. Thus, the proportion of D1R-expressing neurons was approximately 70% in all SP-containing striosomes irrespective of other striosomal markers, approximately 40% in SP-negative striosomes, as low as nearly 30% in striosome-free space, and approximately 50% in the matrix. The proportion of D1R-expressing cells in each of the three types of SP-containing striosomes was significantly higher than that in other compartments composed of SP-negative striosomes, the striosome-free space, Enk-positive islands in matrix, and CB-intense matrix (Figure S8 in the Supplementary material). On the other hand, the proportion of D2R-expressing cells in the striosome-free space (65.0 ± 10.5%) was significantly higher than that in any other compartments. These findings strongly support the fact that the compartment-based map of the striatal internal structure is closely related to the structural organization of the striatum in terms of the direct and indirect parallel streams.

## Discussion

The present study has revealed a site-specific heterogeneity of the striosomes/matrix compartmentalization that represents the uniqueness of the structural organization of the striatum. We focused on this nature to make an internal structure-based map of the striatum, and the obtained map shows consistency with both the topography of corticostriatal projections and the mosaic composition of D1R- and D2R-dominant compartments. Knowledge of objective divisions inside the striatum will greatly facilitate mutual understanding of researchers in different fields with higher resolution for three-dimensional anatomy.

One striking feature found in the composition of the striatum is the presence of large, striosome-free space that surrounds the striosome-rich part of the striatum. This striosome-free space is thought to be a portion of the so-called “dorsolateral sensorimotor area” described in the previous studies in rodents, which has been ambiguous and contains target areas from both primary and associational sensorimotor areas. The absence of compartmentalization, as well as the weak CB-immunoreactivity, clearly demarcated the striosome-free space that was selectively innervated from the primary motor and sensory cortices.

The anatomical segregation of the striosome-free space from the striosome-rich part leads to the intriguing possibility that spiking activities of neurons in the primary motor and sensory areas per se do not directly influence the dopaminergic modulatory system, because innervation of the SNpc from the striatum is conducted only by neurons in striosomes (Gerfen 1985; Fujiyama et al. 2011). In contrast, computation in the associational/limbic cortical areas will influence SNpc activities via activation of striosomes located in the central part of the striatum. This is a reasonable separation in corticostriatal circuitry, because functional roles of associational/limbic cortical areas such as involvement in reward-guided learning and decision-making are closely related to the mobilization of the dopaminergic modulatory system. In contrast, afferents from the primary motor and sensory cortices will transfer information that reflects the final motor output or the centrally converged signals of fine-tuned peripheral senses, both of which may require accuracy rather than dopamine-mediated learning processes during the execution of behaviors.

The five types of striosomes occupied particular positions inside the striatum and showed type-specific compositions of D1R- and D2R-expressing neurons. This means that the map presented here indicates the spatial arrangement of direct pathway-dominant and indirect pathway-dominant striosomal domains. All three types of SP-labeled striosomes that contained a higher proportion (~ 70%) of D1R-expressing neurons were located in the dorsomedial to ventromedial domains in the rostral precommissural striatum and in the medial laminar domain in the postcommissural striatum. On the other hand, two types of SP-weak striosomes, with a proportion of 40% for D1R-expressing neurons, were located in the dorsolateral to ventrolateral domains in the rostral striatum. The lateral decrease in the D1R proportion was accompanied by a lateral increase in the D2R proportion that reached the largest value in the striosome-free space (Fig. 11, see also Figure S8 in the Supplementary material).

These three-dimensional compositions in diverse aspects indicate that site-specific responses of striatal neurons observable during the learning process in vivo occur in the respective domains where constituent neurons have differential molecular and wiring characteristics. In particular, the dorsomedial striatum is responsible for flexible, goal-directed behavioral control, whereas less flexible, reward-insensitive activities leading to habituation occur in the dorsolateral striatum (Yin et al. 2004, 2009; Kimchi and Laubach 2009a, b; Thorn et al. 2010). The former is executed in the medial corticostriatal loop originating from the medial prefrontal areas through the dorsomedial striatum, and the present results indicate that this circuit is suitable to mobilize the dopaminergic regulatory system during reward-guided learning via activation of D1R/direct pathway-dominant striosomes. On the other hand, the dorsolateral striatum is characterized by the richness of the D2R-dominant striosomes, toward which afferents from M2 terminate. Postsynaptic plasticity at D2R-expressing neurons in the dorsolateral striatum has been shown to mediate habit learning through interaction between D2R and transient receptor potential vanilloid 1 channels (Shan et al. 2015). Future physiological studies are expected to elucidate the correlation of marked behavioral differences between the two striatal domains with not only corticostriatal circuitry but also with the differential molecular profiles of the residing neurons.

MOR/SP and MOR-only striosomes, which constituted approximately 60% of all striosomes, were characterized by very weak immunoreactivity for Enk (Figs. 1, 5), which is similar to our previous results (Tajima and Fukuda 2013), though Enk is the endogenous agonist of MOR. In contrast, Enk-rich islands were found in the matrix where MOR-immunoreactivity was weak compared with MOR-labeled striosomes. This apparent discrepancy in the distributions of Enk and its receptor molecule may reflect specific properties in the striosomes/matrix compartmentalization. Weak Enk-immunoreactivity in MOR/SP and MOR-only striosomes suggests that either or both of the number of Enk-positive local collaterals and the content of Enk in individual axon terminals are kept at low levels in such striosomes. If this is the case, a high level of MOR in striosomes may facilitate sensitive responses to slight changes in tissue content of Enk that is kept at a low level in striosomes but could rise when it comes from matriceal Enk-rich islands through volume transmission of this peptide, as suggested to occur as enkephalin surges in the dorsomedial striatum in response to chocolate overeating (DiFeliceantonio et al. 2012). MOR/SP/Enk-labeled striosomes, which were specifically targeted by the agranular insula, will be under the control of mechanisms different from those in MOR/SP and MOR-only striosomes through interaction of high levels of both Enk and MOR.

The most caudal division of the striatum that had not drawn much attention was found to lack compartmentalization but showed a characteristic tri-laminar pattern in composition. Its most medial part was characterized by intense SP labeling and the absence of Enk that corresponds to the “D2 receptor-expressing medium spiny neurons-poor zone” identified in a previous study (Gangarossa et al. 2013). Consistent with the labeling pattern of high SP- and faint Enk-labeling, we confirmed that the vast majority of neurons in this zone expressed D1 receptors (data not shown). In contrast, the neighboring intermediate lamina received inputs from the primary auditory cortex (Fig. 10f, see also Figure S5 in the Supplementary material) and contained an exceedingly low proportion (approximately 20%) of D1R-expressing neurons. The most lateral lamina specifically received inputs from the agranular insula. Moreover, the intermediate lamina is characterized by convergence of dendrites originating from a new type of non-cholinergic large neurons (data will be shown elsewhere). Thus, the most caudal tri-laminar part has many novel properties that have to be determined in future studies. Likewise, the internal structure-based map of the mouse striatum demonstrated here will shed light on broad structure-based researches of the basal ganglia.

## Electronic supplementary material

Below is the link to the electronic supplementary material.

Supplementary material 1 (PDF 13934 KB)
