# Supplementary material for: Striosome-based map of the mouse striatum that is conformable to both cortical afferent topography and uneven distributions of dopamine D1 and D2 receptor-expressing cells
Source: Brain Struct Funct. 2018 Sep 10;223(9):4275–91. doi: 10.1007/s00429-018-1749-3 (PMC6267261; doi:10.1007/s00429-018-1749-3)
Supplement: Supplementary file 1 — Supplementary material 1 (PDF 13934 KB) [file 429_2018_1749_MOESM1_ESM.pdf]

Supplementary materials

Journal: Brain Structure and Function

Article title:

Striosomes-based map of the mouse striatum that is conformable to both cortical afferent topography and uneven distributions of dopamine D1 and D2 receptor-expressing cells

Authors:

Yuta Miyamoto[1], Sachiko Katayama[1], Naoki Shigematsu[1], Akinori Nishi[2], Takaichi Fukuda[1*]

*Corresponding author:

Department of Anatomy and Neurobiology, Graduate School of Medical Sciences, Kumamoto University, 1-1-1 Honjo, Chuo-ku, Kumamoto 860-8556, Japan

E-mail: tfukuda@kumamoto-u.ac.jp

Figure S1

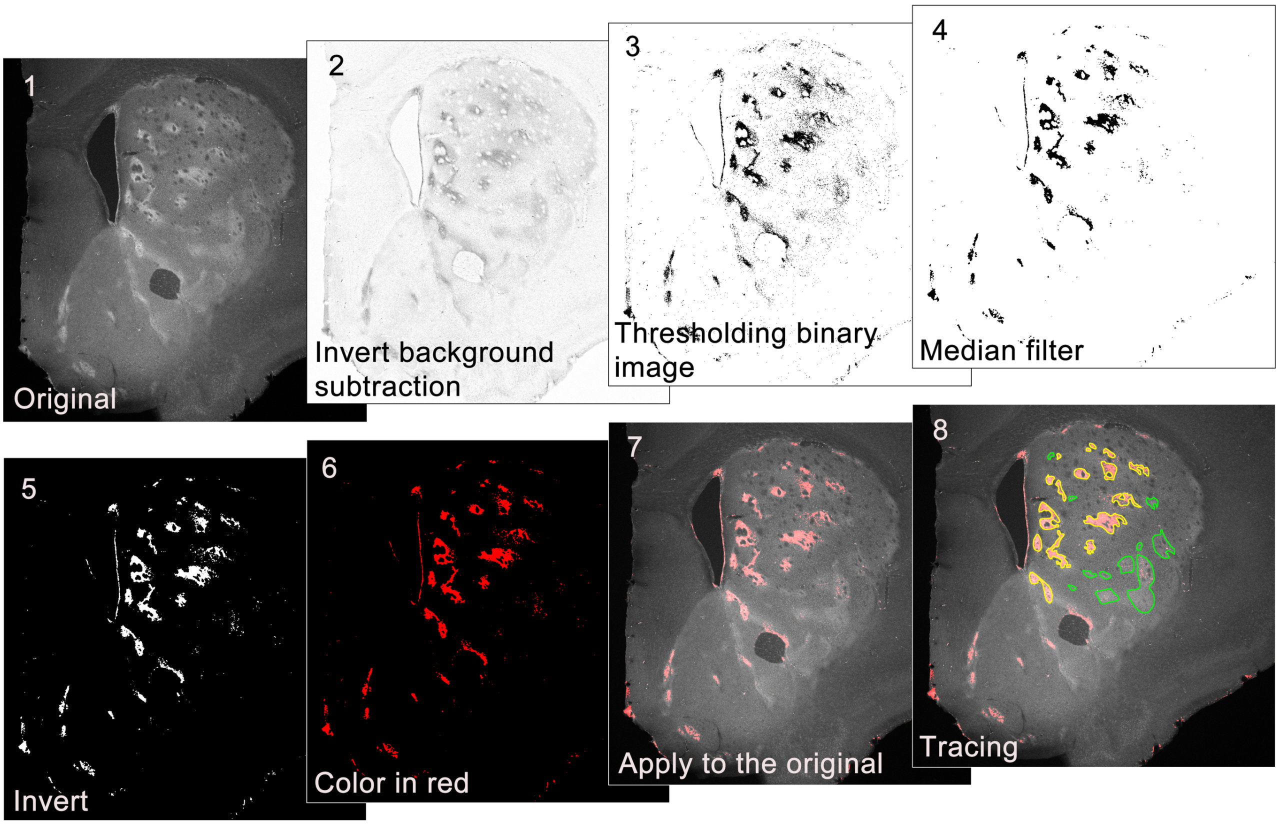

Fig. S1 Procedures for extracting striosomes. Original fluorescence signals acquired in CLSM were processed to facilitate objective tracing of the contours of striosomes as described in detail in the methods section.

Figure S2

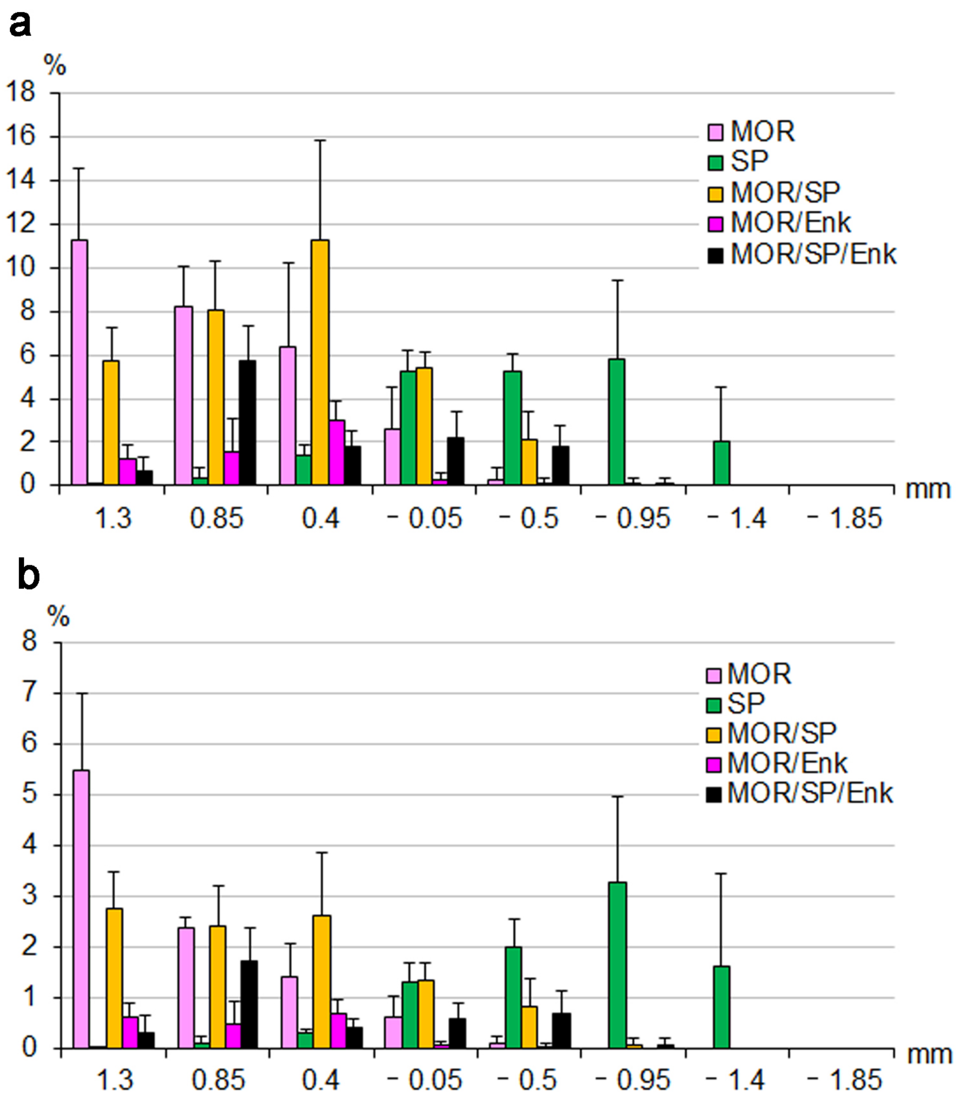

Fig. S2 Distributions of the 5 types of striosomes along the rostrocaudal axis. **a**, The ordinate shows the ratio of the area of each striosomal type at different positions to the total area of all striosomes sampled; thus, the sum of the values of all columns, shown by different colors, is equal to 100%. The ratio at each position was calculated as an average of the measurements obtained in 3 animals. Bars indicate standard deviations. **b**, The ordinate shows the area ratio of each striosomal type, which was defined at each position as the ratio of the summated area of striosomes that belong to the same type in a single section to the area of the striatum of that section. Data are shown as averages of 3 animals.

Figure S3

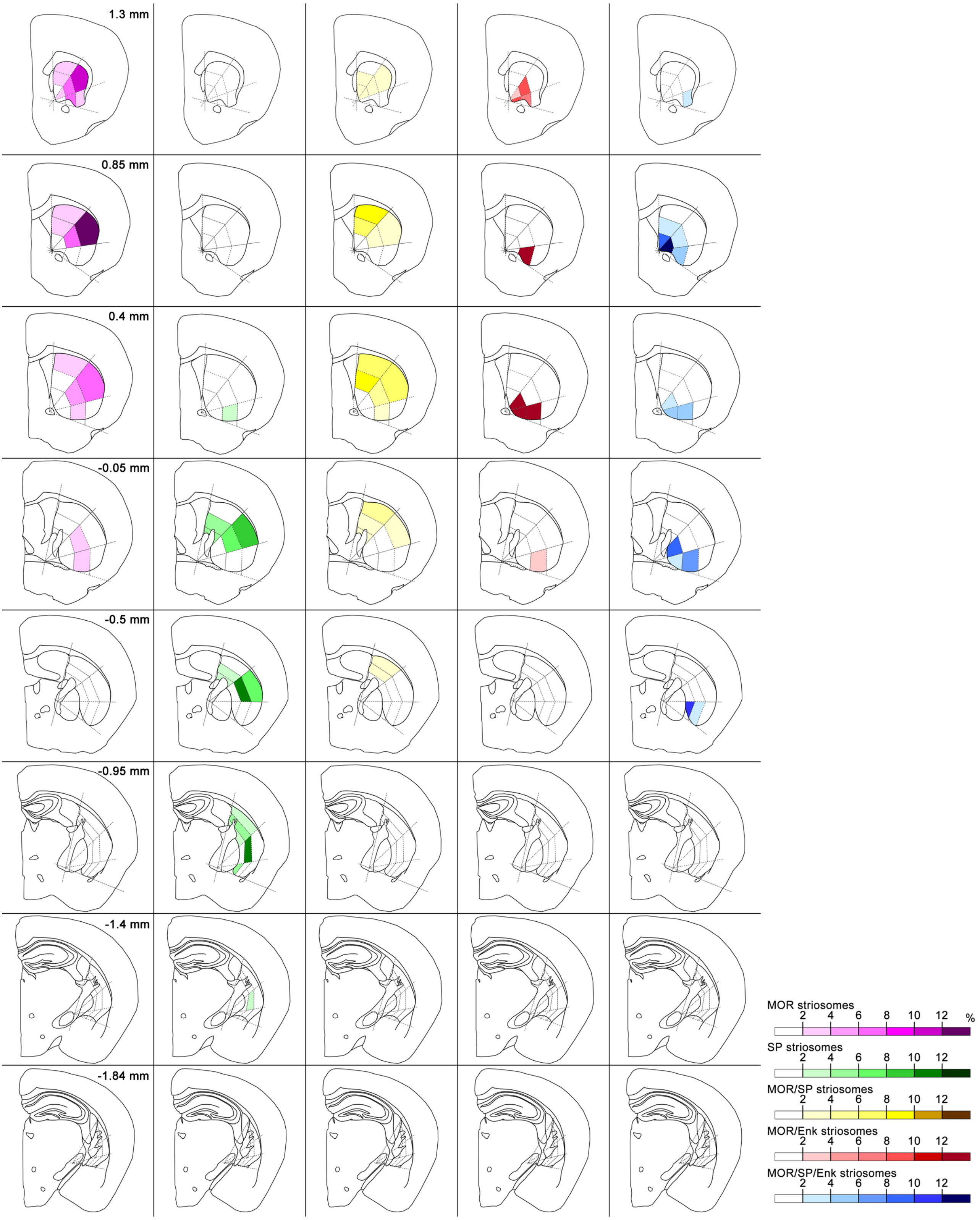

Fig. S3 Segment analysis of the distributions of the 5 types of striosomes. The striatum in individual sections was segmented into 9 subdomains by trisection of both the internal angle and the concentric distance. The

color code for each striosomal type shows the relative proportion of the area of striosomes residing in each subdomain, and the sum of which along the rostrocaudal levels yields 100% for each striosomal type. The value in each subdomain was calculated by averaging the measurements obtained in 3 animals.

Figure S4

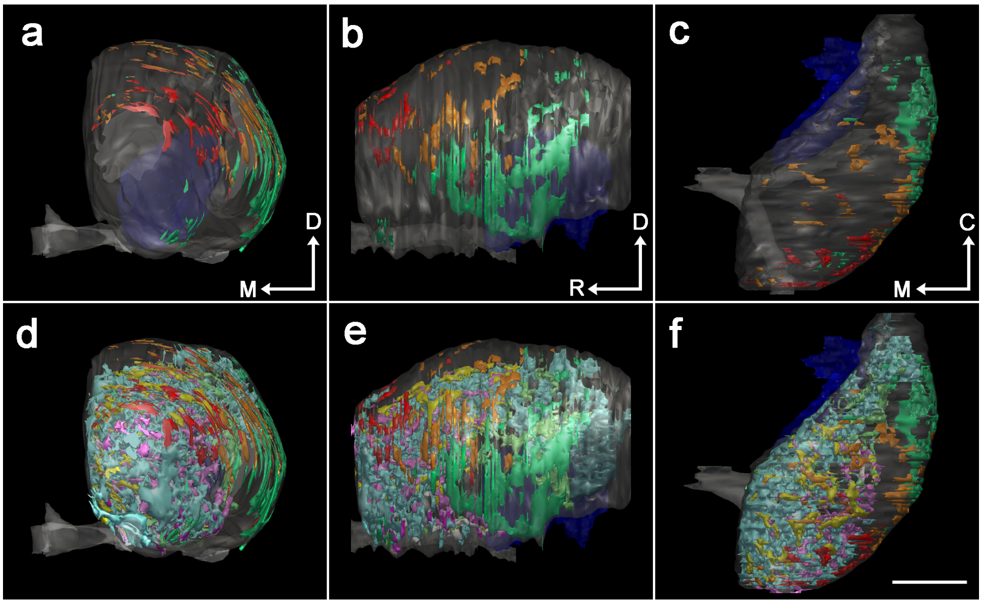

Fig. S4 Subcallosal streaks. Three-dimensional reconstruction of subcallosal streaks labeled for MOR only (red), SP only (green), or both MOR and SP (orange), shown in front (**a**), lateral (**b**), and top (**c**) views. The globus pallidus is shown in dark-blue. **d-f** Combined images of the reconstructions of striosomes, Enk-rich islands, and subcallosal streaks. Scale bar = 1 mm.

Figure S5

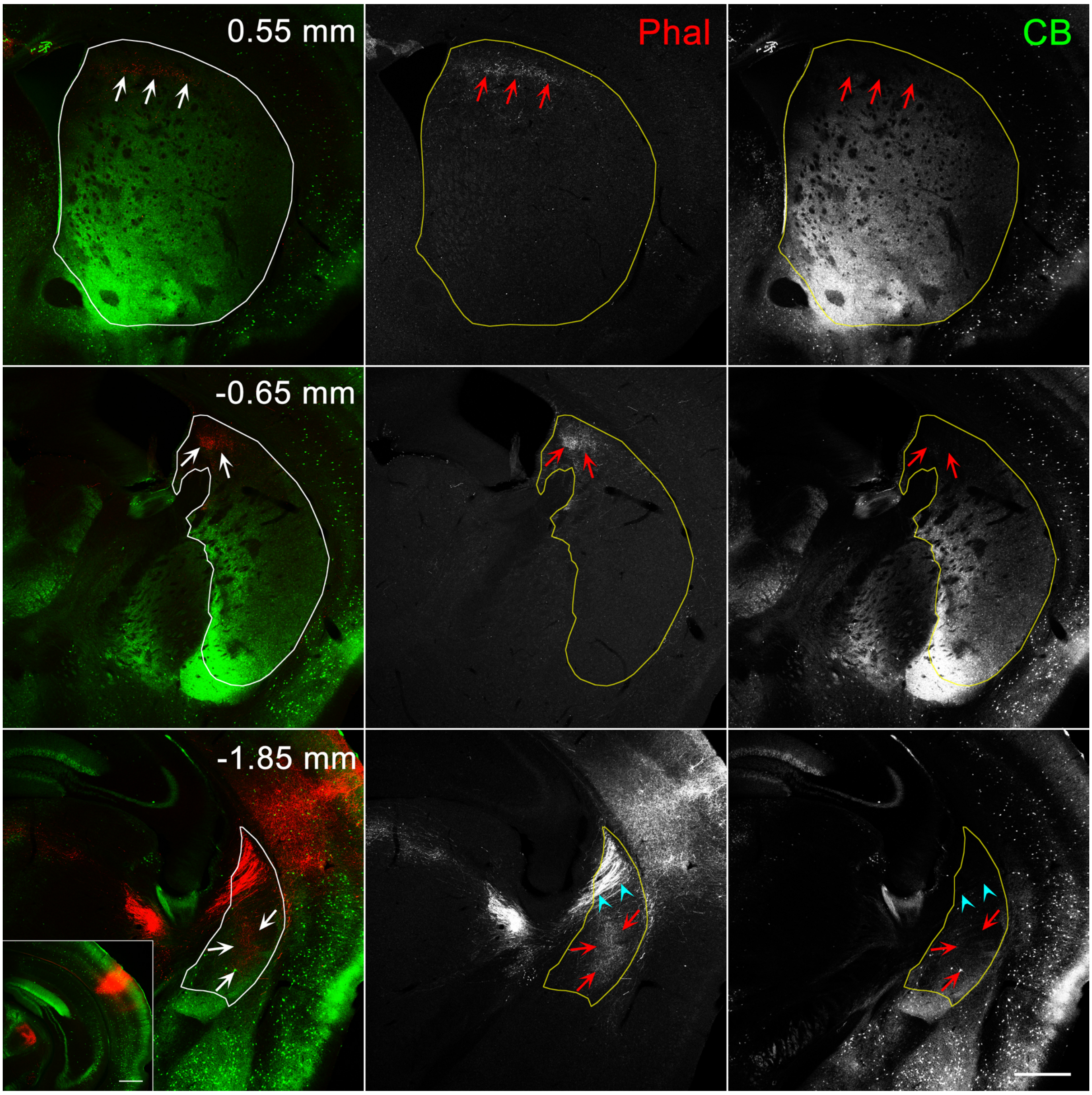

Fig. S5 Innervation of striosome-free space by cortical afferents from the Au1. The site of Phal injection is shown in the inset. Phal-labeled axons and axon terminals are located predominantly in the dorsal, CB-poor/striosome-free space (arrows) at the rostrocaudal positions of 0.55 mm and -0.65 mm. Arrowheads indicate labeling of descending axons in the white matter. The area of innervation at -1.85 mm corresponds to the intermediate band of the most caudal tri-laminar part. Scale bars = 0.5 mm.

Figure S6

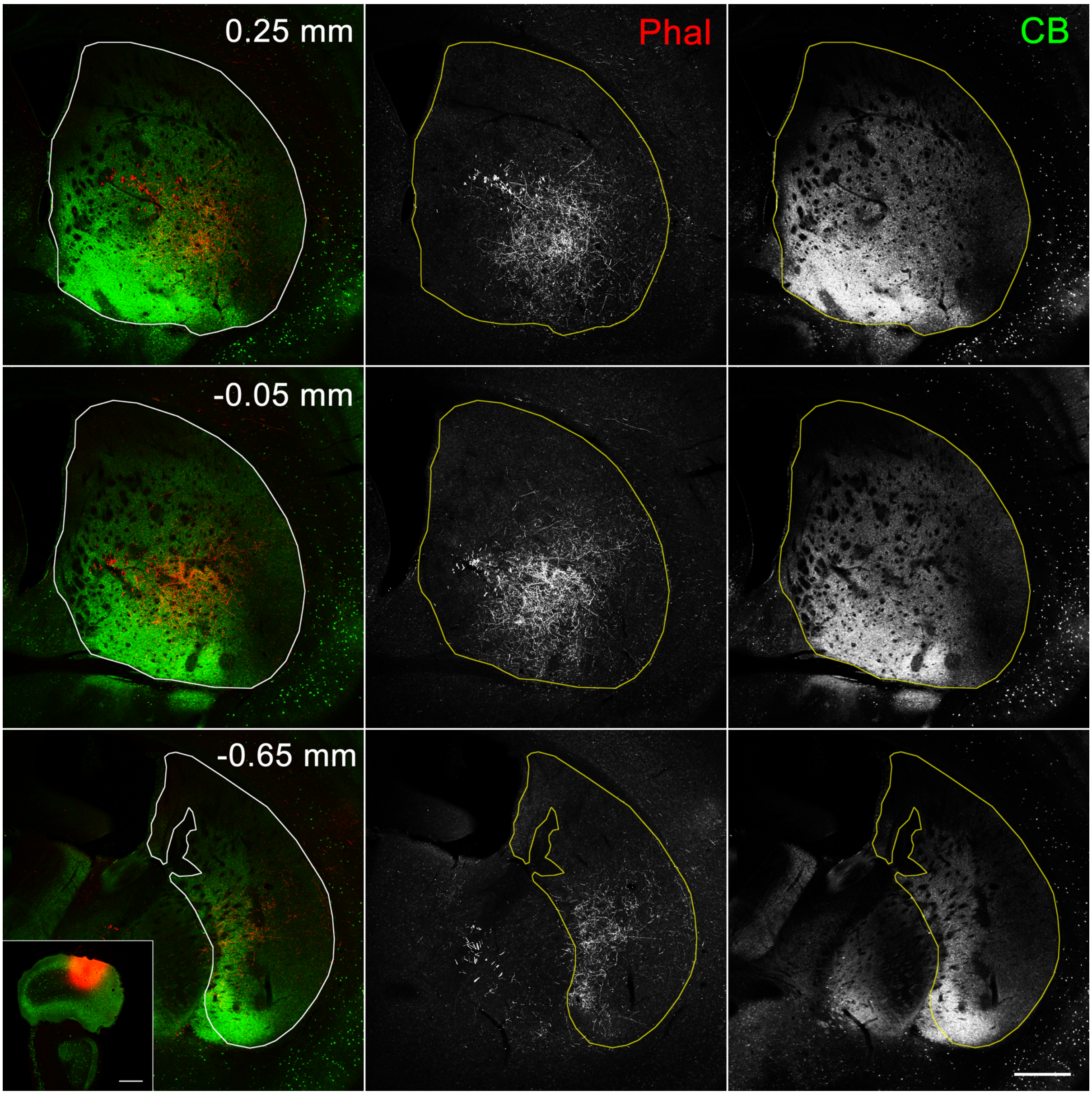

Fig. S6 Innervation of the striosome-rich part by cortical afferents from the FrA. Phal-labeled axons and axon terminals are located predominantly in the ventrolateral part rostrally and in the ventral part caudally, both overlapping with CB-labeled regions. Scale bars = 0.5 mm.

Figure S7

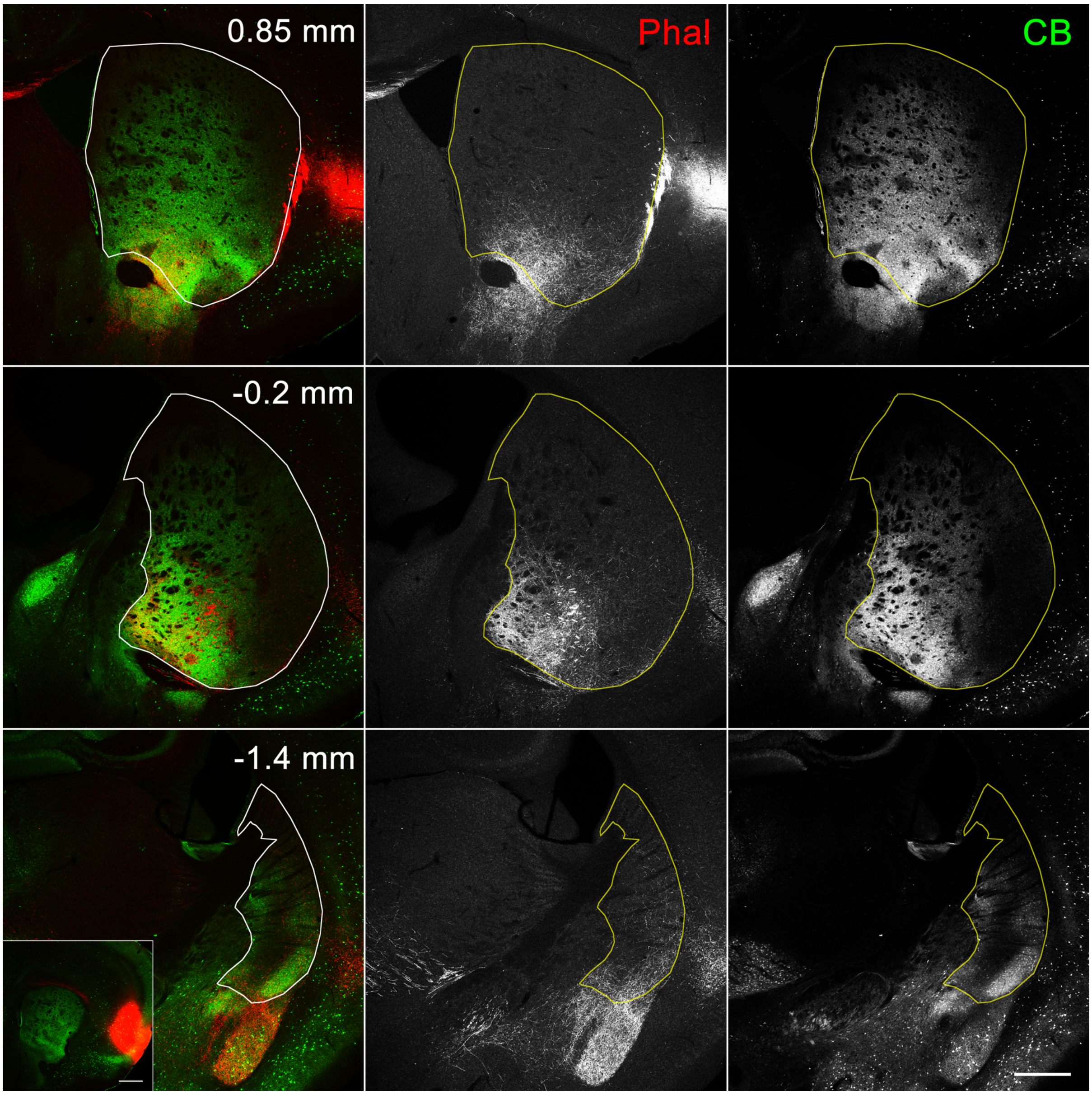

Fig. S7 Innervation by cortical afferents from the agranular insula. Phal-labeled axons and axon terminals are located predominantly in the ventromedial, peripheral part rostrally and in the lateral band of the tri-laminar part caudally, both overlapping with CB-intense regions. Scale bars = 0.5 mm.

Figure S8

D1R	mean ± SD (%)	SP	MOR/SP	MOR/Enk	MOR/SP/Enk	Free space	Enk islands	CB(+) matrix
MOR	42.3 ± 5.3	$p<1.0\times10^{-7}$	$p<1.0\times10^{-7}$	n.s.	$p<1.0\times10^{-7}$	n.s.	n.s.	n.s.
SP	69.0 ± 6.9		n.s.	$p<1.0\times10^{-7}$	n.s.	$p<1.0\times10^{-7}$	0.0000499	0.0000007
MOR/SP	67.9 ± 9.7			$p<1.0\times10^{-7}$	n.s.	$p<1.0\times10^{-7}$	0.0000142	0.0000001
MOR/Enk	42.3 ± 5.1				$p<1.0\times10^{-7}$	n.s.	n.s.	n.s.
MOR/SP/Enk	71.6 ± 8.6					$p<1.0\times10^{-7}$	0.0000005	$p<1.0\times10^{-7}$
Free space	33.2 ± 11.8						0.0000042	0.0002368
Enk islands	52.2 ± 3.2							n.s.
CB(+) matrix	48.9 ± 6.2							

D2R	mean ± SD (%)	SP	MOR/SP	MOR/Enk	MOR/SP/Enk	Free space	Enk islands	CB(+) matrix
MOR	53.9 ± 4.5	$p<1.0\times10^{-7}$	$p<1.0\times10^{-7}$	n.s.	$p<1.0\times10^{-7}$	0.0229744	n.s.	n.s.
SP	30.6 ± 7.1		n.s.	$p<1.0\times10^{-7}$	n.s.	$p<1.0\times10^{-7}$	0.000042	0.0000106
MOR/SP	31.7 ± 9.5			$p<1.0\times10^{-7}$	n.s.	$p<1.0\times10^{-7}$	0.0000125	0.0000025
MOR/Enk	53.3 ± 7.4				$p<1.0\times10^{-7}$	0.022475	n.s.	n.s.
MOR/SP/Enk	28.2 ± 8.8					$p<1.0\times10^{-7}$	0.0000005	0.0000001
Free space	65.0 ± 10.5						0.0000311	0.0001155
Enk islands	47.5 ± 3.0							n.s.
CB(+) matrix	48.6 ± 7.7							

Fig. S8 Diagrams showing the results of the Tukey-Kramer test to compare the ratios of D1R- and D2R-expressing neurons among the compartments. Columns of three types of SP-positive striosomes are shown in green, whereas those of the other compartments are shown in red.
